# Multiscale Co‐reconstruction of Lung Architectures and Inhalable Materials Spatial Distribution

**DOI:** 10.1002/advs.202003941

**Published:** 2021-02-08

**Authors:** Xian Sun, Xiaochuan Zhang, Xiaohong Ren, Hongyu Sun, Li Wu, Caifen Wang, Xiaohui Ye, Peter York, Zhaobing Gao, Hualiang Jiang, Jiwen Zhang, Xianzhen Yin

**Affiliations:** ^1^ Center for MOST and Image Fusion Analysis Shanghai Institute of Materia Medica Chinese Academy of Sciences Shanghai 201210 China; ^2^ Center for Drug Delivery Systems Shanghai Institute of Materia Medica Chinese Academy of Sciences Shanghai 201210 China; ^3^ University of Chinese Academy of Sciences Beijing 100049 China; ^4^ School of Pharmacy East China University of Science and Technology Shanghai 200237 China; ^5^ CAS Key Laboratory of Receptor Research Shanghai Institute of Materia Medica Chinese Academy of Sciences Shanghai 201203 China; ^6^ School of Information Science and Technology University of Science and Technology of China Hefei 230027 China; ^7^ Shanghai Institute for Advanced Immunochemical Studies School of Life Science and Technology ShanghaiTech University Shanghai 200031 China; ^8^ School of Pharmacy University of Bradford Bradford BD71DP UK; ^9^ NMPA Key Laboratory for Quality Research and Evaluation of Pharmaceutical Excipients National Institutes for Food and Drug Control Beijing 100050 China

**Keywords:** dry powder inhalation, fluorescence‐micro optical sectioning tomography, inhaled materials, pulmonary drug delivery, three‐dimensional rendering

## Abstract

The effective pulmonary deposition of inhaled particulate carriers loaded with drugs is a prerequisite for therapeutic effects of drug delivery via inhalation route. Revealing the sophisticated lung scaffold and intrapulmonary distribution of particles at three‐dimensional (3D), in‐situ, and single‐particle level remains a fundamental and critical challenge for dry powder inhalation in pre‐clinical research. Here, taking advantage of the micro optical sectioning tomography system, the high‐precision cross‐scale visualization of entire lung anatomy is obtained. Then, co‐localized lung‐wide datasets of both cyto‐architectures and fluorescent particles are collected at full scale with the resolution down to individual particles. The precise spatial distribution pattern reveals the region‐specific distribution and structure‐associated deposition of the inhalable particles in lungs, which is undetected by previous methods. Overall, this research delivers comprehensive and high‐resolution 3D detection of pulmonary drug delivery vectors and provides a novel strategy to evaluate materials distribution for drug delivery.

## Graphical abstract

Effective deposition of inhaled drug carriers is a prerequisite for intrapulmonary drug delivery systems. The cross‐scale structures of lungs from alveoli to whole organ and region‐specific distributions of the inhalable particles are revealed with an unprecedented resolution by the fluorescence‐micro optical sectioning tomography and image data mining, which provide a novel strategy to evaluate materials distribution for particulate drug delivery.

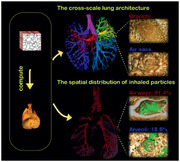

## 1 Introduction

Pulmonary diseases, such as chronic obstructive pulmonary diseases, lower respiratory infections, and lung cancer are listed in the top ten causes of deaths globally with more than 10 million mortality per year.^[^ ^1^ ^]^ Apart from oral administration, the Dry Powder Inhalation (DPI) for pulmonary administration provides an important alternative route for targeted treatment of these pulmonary diseases.^[^ ^2^ ^]^ Meanwhile, materials with the destination of improving the drug deposition at specific sites have been designed as DPI carriers,^[^ ^3^ ^]^ because the depositions of particles carrying drugs at the lesion sites such as alveolar areas associating with lung adenocarcinoma^[^ ^4^ ^]^ and pulmonary fibrosis^[^ ^5^ ^]^ will increase therapeutic effects as well as reduce side effects. Although researches done on exploiting materials with excellent fine particles fraction (FPF) in vitro, the in vivo results are mixed, which means that the gap between in vitro simulations and realistic distribution patterns in vivo is still unfilled. Furthermore, there is a growing demand on understanding the thorough lung architectures, as well as, the accurate distribution patterns in vivo and deposition mechanisms of the inhaled materials at 3D, in situ, and single‐particle level. Thus, a novel strategy that reveals the spatial distribution of inhaled particles precisely at multi‐scale ranging from sub‐micron to the whole lung scale needs to be established.

However, to date, the cross‐scale study of precise lung structure and its inner inhaled particles distribution still faces considerable obstacles. Previous studies have adopted the computed tomography (CT)， magnetic resonance imaging (MRI) and single‐photon emission computed tomography/positron emission tomography (SPECT/PET) technologies to achieve whole‐lung macroscopic imaging,^[^ ^6^ ^]^ whilst they are insufficient to visualize accurate architectures of the terminal bronchioles and alveoli due to the limited spatial resolution (10–50 µm for micro‐CT; 10–100 µm for MRI; 1–2 mm for SPECT/PET).^[^ ^7^ ^]^ In addition, MRI is informed via signals from hydrogen atoms, and thus is incapable of direct imaging the lung structure with massive air content with low response at intense magnetic field.^[^ ^8^ ^]^ For high‐resolution imaging, a tissue‐clearing technique (CUBIC and 3DISCO) combined with light‐sheet microscopy revealed the targeting efficiency of therapeutic antibodies on cancer metastasis in the entire body, which provided an encouraging approach to investigate the cross‐scale tissue structures in the future.^[^ ^9^ ^]^ Furthermore, the in vivo imaging system (IVIS) has been utilized as a routine method to ascertain the particles distribution at 2D level.^[^ ^10^ ^]^ Dynamic X‐ray phase‐contrast imaging with temporal resolution was used to observe micro‐droplet deposition during and immediately after intrapulmonary delivery.^[^ ^11^ ^]^ Although these studies provided the distributing situation of inhaled particles at whole‐lung scale, observing single particles at the alveolar area remains as a major challenge. To reveal particles distribution at alveolar areas with high resolution, a tissue clearing technique (Clear^T2^) combined with laser scanning confocal microscopy (LSCM) has been applied to show the intrapulmonary distribution of aerosolized drugs in selected small local regions.^[^ ^12^ ^]^ Generally, there remains a lack of detection on the accurate spatial distribution of inhaled particles at the whole‐lung scale with subcellular resolution.

**Scheme 1:**
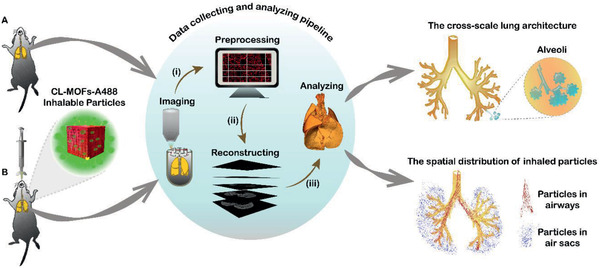
Schematic illustration of the strategy to coreconstruct the multiscale lung architectures and the inhaled particles distribution taking advantage of the micro‐optical sectioning tomography (MOST) and fluorescence‐micro‐optical sectioning tomography (f‐MOST) system Specifically, the mouse lungs B) with or A) without inhaled particles were extracted followed by staining and dehydration. i) Then the resin‐embedded mouse lung was fixed on a base for imaging. The voxel size is 0.35 × 0.35 × 1 µm for MOST system and 0.35 × 0.35 × 2 µm for f‐MOST system. ii) The gathered mosaic images were preprocessed, which included seamless stitching, luminance non‐uniformity correction, and image defect removal. iii) The cross‐scale lung architectures were reconstructed by overlapping about 80 000 coronal images. Finally, image segmentation, three‐dimensional (3D) structure rendering and morphological characterization were performed using Amira Software for the qualitative analysis of the lung structure (airways, arteries, veins, and alveoli) and quantitative computing of the inhaled particles’ deposition in different areas.

In this study, high‐precision cross‐scale visualization of entire lung anatomy was acquired by the advanced Micro‐Optical Sectioning Tomography (MOST) system coupled with whole lung Nissl‐staining (**Scheme** **1**A). In addition, Alex Fluorescence 488‐labelled cross‐linked *γ*‐cyclodextrin metal‐organic frameworks (CL‐MOFs‐A488) were synthesized and characterized as model particles, and dual‐color and lung‐wide datasets were acquired by fluorescence‐micro‐optical sectioning tomography (f‐MOST) system following intrapulmonary administration (Scheme 1B). More importantly, the sophisticated architectures of the mouse lungs and the region‐specific distribution patterns and structure‐associated deposition of the inhalable particles in lungs were revealed simultaneously for the first time, providing new approaches for understanding the deposition behavior and distribution of inhaled materials in vivo. This research will provide precise anatomical data for in vitro simulations and contribute to fill the gap between in vitro and in vivo analyzing results, which will facilitate the clinical translations of novel inhalable DPI vehicles.

## 2 Results

### 2.1 Whole‐Lung Airway Tree and Vascular Systems

**Figure 1:**
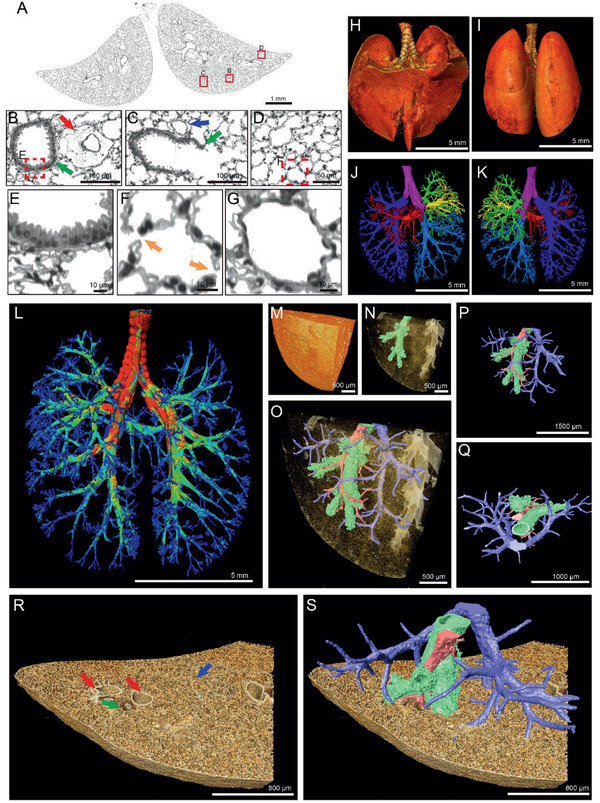
Tracheal tree and vascular systems corendering. A) The slice of lung gained by Nissl‐staining and MOST system. B–D) Enlarged image of the areas marked in red box in (A). E) The surface structure of bronchi obtained from the data corresponding to the red box region in (B), the grey value range for trachea segmenting was 110 to 255. F) The enlarged alveolar structure, the yellow arrows indicated the gaps among alveolar epithelial cells. G) The capillary in alveolar area. H,I) The 3D rendering of mouse lungs with Nissl‐staining in ventral and dorsal views, respectively. J,K) The 3D visualization of the tracheal tree corresponding with (H) and (I). The different colors revealed the different lung lobes. L) The tracheal tree colored by inner diameter. M) A small region of lung, cropping from blue box in (I). N) The bronchioles in lungs. O) The co‐reconstruction of airways, veins, and arteries. P,Q) The obverse and top views of extracted vasculature systems. R,S) The airways, veins, and arteries in pulmonary parenchyma. Red arrows: arteries; green arrows: airways; blue arrows: veins.

The MOST system combined with Nissl‐staining was utilized to visualize the sophisticated structure of lung. **Figure** **1**A displayed the coronal image of mouse lung after preprocessing. In line with the conventional HE staining (Figure S1, Supporting Information), coronal images created by the MOST system were reliable to reconstruct the lung architectures. The specific structures of bronchi, terminal bronchioles, alveoli, arteries, veins, and capillaries were distinguished based on the diversity of grey levels and morphological features. The surface of bronchi was uneven with a grey value range from 110 to 255 (Figure 1B, green arrow, & Figure 1E). These specific features disappeared in the alveolar duct (Figure 1C, green arrow). The alveolar epithelial cells formed the acini network (Figure 1D), but spread discretely with spacious gaps (Figure 1F, yellow arrows). In contrast, the capillary walls were relatively compact (Figure 1G). The arteries (Figure 1B, red arrow) had thick walls compared with veins (Figure 1C, blue arrow). The whole lung images were shown as Figure 1H,I. The multiscale architectures from trachea to terminal bronchioles were reconstructed taking advantage of the distinctive grey value of airway (Figure S2A,B and Movie S1, Supporting Information). The repeated tiers of branching in the rendered airway tree terminated at alveolar ducts (Figure S2C, Supporting Information). The intact lung structures including all generations of airways and acini network were visualized and segmented (Figure 1J,K). As there is no unified airways grading methods for mice, if referring to the classifications for human, this method can visualize the generations from G0 (trachea) to G23 (alveolar sacs) based on Weibel A model, and stages from S1 to S7 according to NGI model. The internal airways in five lung lobes are formed by repeated branching, and the orientations of bronchi and bronchioles were various. The airway tree colored by inner diameter was shown in Figure 1L. At a local region, the 3D structure of bronchioles, small arteries and veins were co‐reconstructed simultaneously (Figure 1M–O and Movie S2, Supporting Information). The lung structure was complex, containing many tubes systems (airways: green; arteries: red; veins: blue) in the sponge‐like texture created by millions of air‐filled alveoli (Figure 1R,S). The extracted bronchioles and vessels reflected that the arteries serving the bronchioles and the veins were relatively distant (Figure 1P). The inner diameter of the artery as shown was smaller than that of the bronchiole and vein (Figure 1Q). Moreover, the images of the veins revealed more branching than arteries (Figure 1P, blue).

### 2.2 Three‐dimensioal Morphological Features of Airways and Vascular Systems

**Figure 2:**
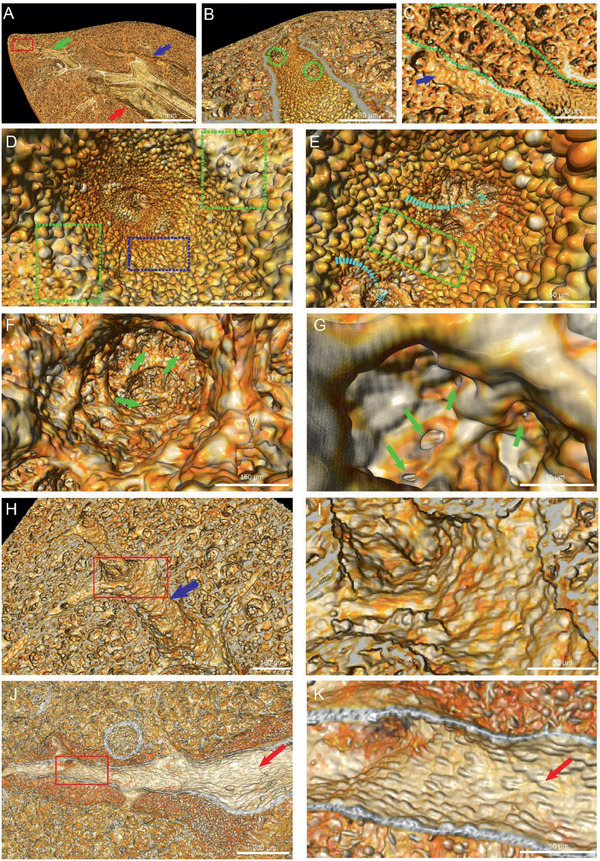
High‐resolution morphological analyses of airways and vascular systems. A) The local cross profile of lung containing bronchioles (green arrow), veins (blue arrow), arteries (red arrow). B) The enlarged image of the terminal bronchiole indicated by green arrow in (A). The dotted green circles reflected the outlets on a terminal bronchiole. C) An enlarged view of the area indicated by the red square in (A), demonstrating the structure of terminal outlet and alveoli duct. The blue arrow points at a vein. D,E) The inside view of airway walls. The spaces among ‘humps’ at bifurcations (green dotted boxes) were larger than flat areas (blue dotted boxes). Cyan arrows in (E) showed the outlets of terminal bronchioles. F) The sponge‐like air sacs area. Alveoli connected with each other through holes (green arrows). G) Adjacent alveoli communicated through pores of Kohn (green arrows). H) The veins in acinus (blue arrow). I) Detailed view of venous surface corresponding to the red square area in (H). J) The arteries (red arrow). K) An enlarged view of arterial surface cropping from the red box in (J). The protuberances (red arrow) showed orientation.

A portion of the lung containing distinctive bronchioles (green arrows), arteries (red arrows), and veins (blue arrows) was segmented (Figure S2D–F and Movie S2, Supporting Information). There were at least five outlets in a terminal bronchiole at the length of 1 mm (**Figure** **2**A,B). These air sacs were located sparsely around the respiratory bronchioles and connected with each other, forming a huge spongiform network (Figure 2C). In addition, a large number of hill‐like architectures (with the diameter of around 20 µm) covered the inner wall of bronchioles (Figure 2D and Figure S2G,H, Supporting Information). These surface features (humps), were always perpendicular to the surface of the airways, with a reducing distribution density at bifurcations and direction‐changing areas of airways. At these areas, the space between humps was enlarged (Figure 2D,E, dotted green boxes). The disappearance of these humps coincided with the end of airways and the start of alveolar ducts, the epithelium‐lined tubules. The diameter of the alveolar entrance was about 200 µm and the walls of acinus were smoother than that of airways (Figure 2F). The alveoli in a whole lobe were interconnected by holes (Figure 2F, green arrows). Furthermore, there were apertures known as the pores of Kohn between two adjacent alveoli.^[^ ^13^ ^]^ As shown in Figure 2G (green arrows), these alveolar apertures were circular with a diameter of 1–5 µm. The inner walls of arteries and veins were rough (Figure 2J). The protuberances on arterial endothelia showed orientation, corresponding with the direction of blood flow (Figure 2K) whilst this orientation was not observed on the internal surface of veins (Figure 2H,I and Figure S2I,J, Supporting Information).

### 2.3 Fluorescence‐Labeled Cross‐Linked *γ*‐Cyclodextrin Metal‐Organic Frameworks Acting as Inhalable Model Particles for Tracking

**Figure 3:**
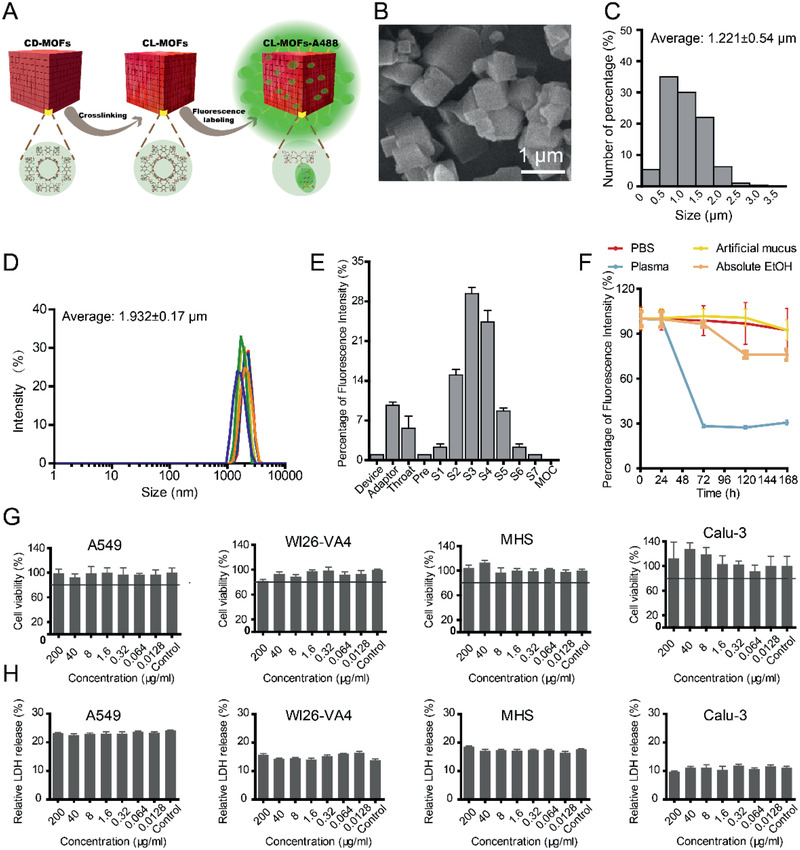
Synthesis and characterization of Alex fluorescence 488‐labelled cross‐linked γ‐cyclodextrin metal‐organic frameworks (CL‐MOFs‐A488) particles. A) The schematic synthesis of CL‐MOFs‐A488 particles. The red cubic matrix represented γ‐cyclodextrin metal‐organic frameworks (CD‐MOFs) and cross‐link CD‐MOFs (CL‐MOFs), respectively. The CD‐MOFs were prepared as templates to produce CL‐MOFs tethered by diphenyl carbonate (DPC). Fluorescent agent of Alexa fluor 488‐NHS was conjugated on CL‐MOFs to obtain CL‐MOFs‐A488 for tracking. B) SEM was applied to get the morphology of CL‐MOFs‐A488. C) The size distribution of CL‐MOFs‐A488. D) The hydrodynamic size of CL‐MOFs‐A488 E) The next generation impactor (NGI) was applied to test the aerodynamic particle size and in vitro deposition of CL‐MOFs‐A488. At the flow rate of 65 L min^−1^ (P1 = 4 kPa), the cutoff particle sizes of NGI 1–7 segments were 7.73, 4.20, 2.68, 1.63, 0.90, 0.52, and 0.32 µm, respectively. Up to 79.0 ± 2.8% of CL‐MOFs‐A488 deposited at stages 2–6 (*n* = 3). F) The changes of the fluorescent intensity of CL‐MOFs‐A488 in four media detected by in vivo image system over 7 days (*n* = 5). G,H) Cellular viability of CL‐MOFs‐A488 on four cell lines (A549, WI26‐VA4, MHS, and Calu‐3) detected by G) CCK‐8 investigations and H) LDH release assays (*n* = 6). The data were processed by GraphPad Prism 5 and presented as means ± s.d.

For studies of inhaled particles distribution throughout the mouse lungs, Alex fluorescence 488‐labelled cross‐linked γ‐cyclodextrin metal‐organic frameworks (CL‐MOFs‐A488) with uniform cubic shape were synthesized as model inhalable particles. The synthesis procedure was illustrated in **Figure** **3**A. Scanning electron microscopy (SEM) and statistical analysis showed that CL‐MOFs‐A488 were homogeneous cubes with the size of 1.221 ± 0.54 µm (Figure 3B,C). The hydrodynamic size measured by dynamic light scattering was 1.932 ± 0.17 µm (Figure 3D). The average zeta potential of CL‐MOFs‐A488 was −32.37 ± 0.42 mV, suggesting the fine dispersibility in water (Figure S3A, Supporting Information). The aerodynamic size of inhaled particles is one of the critical factors to control its deposition behaviors,^[^ ^14^ ^]^ and the Next Generation Impactor (NGI) was adopted to characterize the properties of powders.^[^ ^15^ ^]^ The FPF value of CL‐MOFs‐A488 particles was 79.0 ± 2.8% (Figure 3E and Figure S3E, Supporting Information) and the mass median aerodynamic diameter (MMAD) of CL‐MOFs‐A488 was 2.912 ± 0.085 µm, indicating an excellent aerodynamic size distribution of CL‐MOFs‐A488 as inhaled particles. Geometric standard deviation (GSD) was used to reflect the dispersion of the aerodynamic particle size distribution. The GSD of CL‐MOFs‐A488 was 1.624 ± 0.013, suggesting a narrow aerodynamic size distribution. The TGA analysis indicated that the decomposition temperature of CL‐MOFs‐A488 was 314 °C, revealing good thermal stability (Figure S3B,C, Supporting Information). The linear correlation with the regression coefficient of 0.9966 (Figure S3D, Supporting Information) indicated that the fluorescence intensity could be utilized for quantitative evaluation of CL‐MOFs‐A488. Then, the stabilities of fluorescent intensity and morphology were investigated. Figure 3F showed that the PBS and artificial mucus had minor effects on the fluorescent attenuation. Whilst absolute ethyl alcohol can weaken the fluorescent intensity, and the presence of plasma led to a major reduction in fluorescent signals with a fall to 28% at 72 h. The morphology features captured by LSCM were displayed by Figure S4A. The results demonstrated that the fluorescent signals remained square shape if present, indicating the maintenance of cubic form of CL‐MOFs‐A488. If the fluorescence had vanished and CL‐MOFs‐A488 had not degraded, there would have particles without fluorescence. However, this phenomenon was not observed (Figure S4B, Supporting Information). Accompanied with the major decrease of fluorescent intensity in plasma at 72 h (Figure 3F and Figure S3F, Supporting Information), the cubic form of CL‐MOFs‐A488 disappeared (Figure S4A, Supporting Information). From these results, it is concluded that the disappearance of fluorescence signals represented the degradation of CL‐MOFs‐A488. Furthermore, CL‐MOFs‐A488 particles were delivered into mouse lungs. Data collected by the IVIS illustrated that the majority of CL‐MOFs‐A488 degraded at 72 h (Figure S3G, Supporting Information). The cyto‐compatibility of CL‐MOFs‐A488 was confirmed on Type I alveolar epithelial cells (WI26‐VA4),^[^ ^16^ ^]^ Type II alveolar epithelial cells (A549), alveolar macrophages (MHS) and Tracheal epithelial cells (Calu‐3). The cell counting kit‐8 (CCK‐8) assay and lactate dehydrogenase (LDH) release assay confirmed the biocompatibility of CL‐MOFs‐A488 (Figure 3G,H). The internalization of CL‐MOFs‐A488 was also explored in A549, WI26‐VA4, and MHS cell lines. CLSM observations revealed that CL‐MOFs‐A488 aggregated on the surface of A549 cell membranes (Figure S5A, yellow arrows, Supporting Information), and taken up by WI26‐VA4 and MHS (Figure S5A, white arrows, Supporting Information). The evaluation based on fluorescent signals clarified that CL‐MOFs‐A488 were primarily taken in and cleared by MHS in the alveolar area (Figure S5B,C, Supporting Information). These findings were confirmed by immunofluorescence investigation (Figure S6, Supporting Information). However, results from enzyme‐linked immunosorbent assay indicated that concentration‐dependency was occurring for CL‐MOFs‐A488 to stimulate inflammatory response (Figure S5D–F, Supporting Information).

### 2.4 The Distribution of Inhaled Particles at Whole Lung Scale

CL‐MOFs‐A488 were delivered into mouse lungs by endotracheal intubation with an otoscope to visualize the throat (Figure S7A–D, Supporting Information). The percentages of delivered dosage with different dosages (1 and 2 mg) and air volumes (0.5, 1, and 2 mL) were investigated thoroughly. Regarding the mice survival state, the administration of 1 mg particles with 1 mL air were administered to mouse lungs (Figure S7F, Supporting Information). At this condition, the net delivered particle percentage was 84.35 ± 0.05% (Figure S7G, Supporting Information). The distributions of CL‐MOFs‐A488 in lungs were detected by IVIS as a contrast. IVIS provided an indication of the distribution of CL‐MOFs‐A488, like a cloud on the lungs at 2D level, but did not reveal any detailed information such as the accurate position and the deposition ratio in different areas (Figure S7E, Supporting Information). To eliminate this issue, the mouse lungs with inhaled CL‐MOFs‐A488 were prepared for f‐MOST imaging via the process shown in Figure S8A–C, Supporting Information. The coronal image was shown as Figure S8D, Supporting Information. The surface of the trachea was composed of pseudostratified ciliated columnar epithelial cells with high density of nucleus compared to other regions. The serried distribution of nuclei on the surface of the trachea generated the distinctive areas (Figure S8E, Supporting Information).

**Figure 4:**
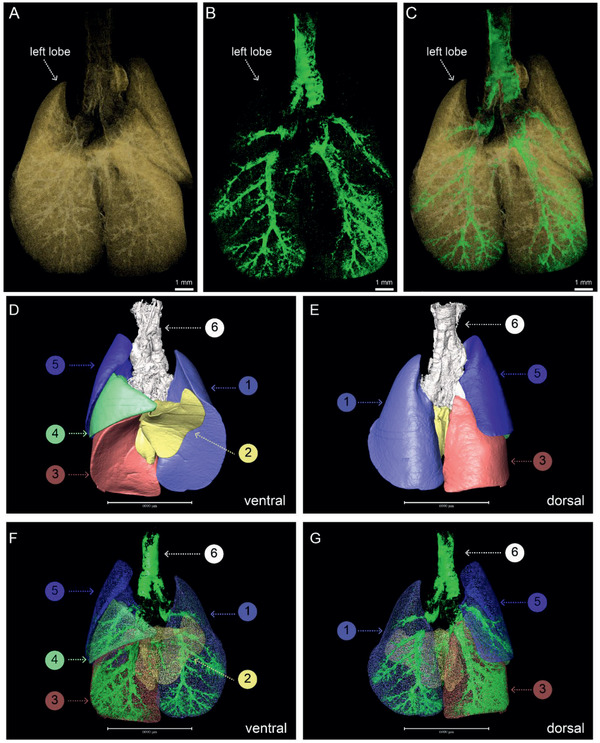
The distribution patterns of inhaled particles at full lung scale. A) The perspective of internal trachea of lung. B) The spatial distribution of CL‐MOFs‐A488 in mouse lung. C) Merged images of whole‐lung structure and CL‐MOFs‐A488 distribution within. D,E) The mice lung were divided into six parts including 1) left lobe, 2) accessory lobe, 3) right caudal lobe, 4) right middle lobe, 5) right cranial lobe, and 6) extrapulmonary trachea. F,G) The deposited particles in different parts. The deposition fractions were 27.00% (left lobe), 2.50% (accessory lobe), 47.05% (right caudal lobe), 3.21% (right middle lobe), 4.70% (right cranial lobe), and 15.52% (extrapulmonary trachea).

**Figure 5:**
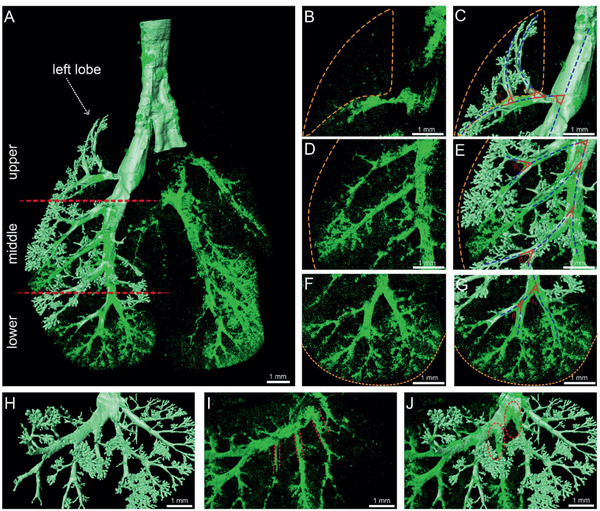
The CL‐MOFs‐A488 particles distribution in the left lobe. A–G) The particles distribution and local airways in B,C) upper, D,E) middle, and F,G) lower level. The blue dashed lines indicated the tracheal and bronchi orientation. The yellow dashed line indicated the periphery of the lung lobe. The red line demonstrated the angles between airways. The values were demonstrated in Figure S13, Supporting Information. The enlarged view of H) airways tree, I) particles, and J) merged image. The dashed red lines represented border of bronchi. The red dashed circle hinted out the bifurcations.

The deposition sites of micron‐sized particles or clusters, which influence the therapeutic effect, are critical. The CL‐MOFs‐A488 particles and PI‐based lung structure were overlaid (**Figure** **4**A–C and Movie S3, Supporting Information) to illustrate the distribution patterns of inhaled particles. The particle distributions were varied in different regions of the lungs. The particles distributed along the respiratory tracts, and the majority of particles adhered on the surface of airways (Figure 4B). Only 18.6% of inhaled particles was delivered to the acini areas. In addition, the lung was divided into six parts including five lobes and extrapulmonary trachea (Figure 4D,E). The quantitative results revealed that nearly half of the particles (47.05%) deposited at the right caudal lobe. The percentage of particles in left lobe ranked the second (27.00%) followed by extrapulmonary trachea with 15.52% (Figure 4F,G). The distribution ratio of deposited particles in right‐left lung was 2.13, indicating that the volume of deposited particles in right lung was double to that in left lung. Meanwhile, the diameter of right extrapulmonary bronchi was much larger than that of left ones (Figure S9D, Supporting Information) indicating the larger ventilation volume of the right lobes. Considering the diversity of lung lobes’ volume,the data were normalized by using particles’ volume divided by lung lobes’ volume. The particle density in each lobe was 1.39 (left lobe), 0.53 (accessory lobe), 3.40 (right caudal lobe), 0.47 (right middle lobe), and 0.65 (right cranial lobe). If the particles deposited equally in each lung lobe, then the particle density should be similar. However, there are huge diversities among five lobes. The particle density in right caudal lobe was almost 7.5 times that of right middle lobe. The densities of particles in sacs of same lobes were also varied (**Figure** **5**A–G). The particles in lower (Figure 5F,G) and middle areas (Figure 5D,E) were denser than that in upper ones (Figure 5B,C). For the bronchi and bronchioles, the orientations of bronchi and bronchioles in the upper and lower levels were opposite and the angles between bronchi were different (Figure S9A–C, Supporting Information). The bronchioles were upward in upper part (Figure 5C), and the angles of *α*_1_ was 47.7°, the values for *β*_1_ and *β*_2_ were 70.9° and 76.4°, respectively. In contrast, the bronchioles in middle and lower level were downwards (Figure 4E,G) and the angles for *α*_2‐5_ were similar or even larger than that for *α*_1,_ and the angles of *β*_3_ were closer (72.6°) to *β*_1_ and *β*_2._ The angles of *β*_4_ and *β*_5_ were 34.8° and 53.0°, half of that determined for *β*_1–3_. In addition, these particles were more likely to deposit at the down side of bronchi (Figure 4H,I) and the bifurcation of airways (Figure 4J).

### 2.5 The Aggregated Particles on the Surface of Respiratory Tracts

**Figure 6:**
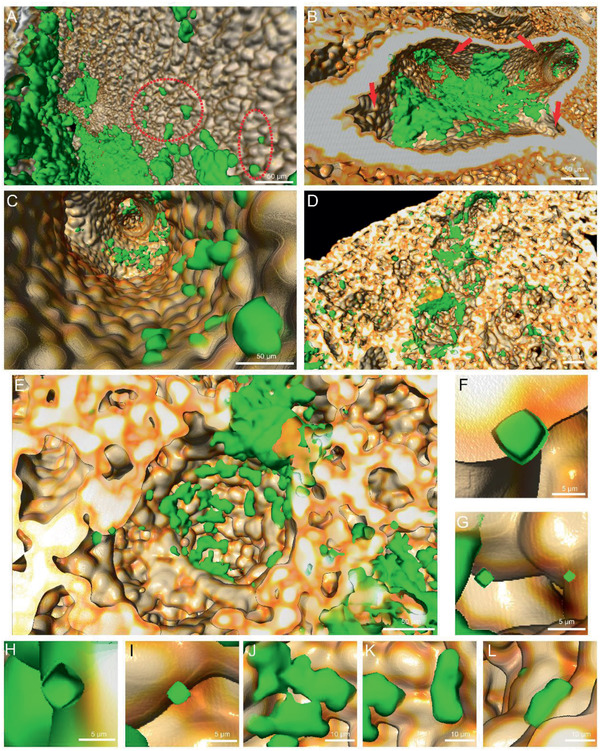
Aggregated particles distributed in all generations of airways. The location of inhaled CL‐MOFs‐A488 on the A) surface of tracheal, B) bifurcations among bronchi, C) bronchioles, D) air sacs, and E) individual alveoli. F–L) The single particles F–I) and small aggregations J–L) in alveoli.

The projection of lung along the bronchiole was shown as Figure S10A, Supporting Information. The aforementioned high brightness structure extended to terminal bronchioles and disappeared at alveolar duct (Figure S10B, Supporting Information). In addition, aggregation of particles was observed at the outlet of the terminal bronchioles (Figure S10C, Supporting Information), and the particles adhered on the surface of bronchi (Figure S10D, Supporting Information). The displayed transecting projections revealed that CL‐MOFs‐A488 particles gathered in alveolar ducts (Figure S10E, Supporting Information), with individual particles being in the alveolar sacs (Figure S10F, Supporting Information). A 100 µm‐thick section including three specific structures was chosen to display specific particle distribution in different structures (Figure S10G, Supporting Information). CL‐MOFs‐A488 particles clustered in trachea and bronchioles. Whilst the particles in alveoli were sparse as only small aggregates or single particles in alveolar sacs. **Figure** **6** showed the location of CL‐MOFs‐A488 on the surface at different airway generations. In the upper respiratory tract, there was an extensive deposition of particles (Figure 6A), and some small aggregates were embedded in the uneven structure of the trachea surface (Figure 6A red circle). In addition, more particles were evidenced at the bifurcations among bronchi (Figure 6B). Aggregates were found in bronchioles with a diameter of 100 µm (Figure 6C). The clusters of CL‐MOFs‐A488 particles in air sacs (Figure 6D,E) were small (less than 20 µm) (Figure 6J–L). There were also single particles with a cubic shape in alveoli (Figure 6F–I). The endoscopic views of inhaled particles in bronchioles and alveoli were demonstrated as Movie S3, Supporting Information.

## 3 Discussion and Conclusion

First, Nissl‐staining was found to be applicable for lung structure rendering. The slices generated by the MOST system manifested the distinctive structures of trachea and bronchioles, confirming the feasibility of the MOST system for characterization of the structural detail of lungs. Nissl‐staining was originally developed for morphological and pathological observations of neurons.^[^ ^17^ ^]^ Nissl reagents were basic dyes with high affinity to chromophilic substances in cells, such as nucleus and Nissl bodies consisting of rough endoplasmic reticulum (RER). The pseudostratified ciliated columnar epithelial cells and goblet cells are distributed on the surface of trachea. This not only resulted in darker staining of airways surfaces but also the specific irregular topography with ‘humps’ covering on the surface.^[^ ^18^ ^]^ Moreover, tracheal epithelial cells, with the function of mucus secretion, contained RER, which also contributed to the deeper staining. In this context, the whole‐lung airway tree (from trachea to terminal bronchioles even the acini network) and vascular systems were rendered simultaneously with Nissl‐staining, and the microstructures of terminal bronchioles, vessels, and alveoli were analyzed at the 3D level, benefiting from the high resolution of the MOST system. It was interesting to find that there were blood flow‐oriented protuberances on arterial endothelia, caused by the high shear stress in arteries leading to the endothelial cells becoming elongated and reshaped to fusiform.^[^ ^19^ ^]^ The cell nucleus shifted to the side corresponding with the blood flow direction. Thus, the surfaces of the arteries contained protuberances with unified orientations. This phenomenon was not observed on the veins, since the shear stress on venous endothelium is relatively low, and the endothelial cells are round in shape. Compared with other techniques, our approach directly observed the normal tissue without labeling the airways and vessels with any fluorescent proteins/molecules and contrast agents.

CL‐MOFs‐A488 particles with mono‐size distribution and uniform shape were fabricated as model inhalable particles. The prior study has created cholesterol modified CD‐MOFs particles with a FPF of 30.60 ± 0.76% for intrapulmonary drug delivery.^[^ ^20^ ^]^ Here, CD‐MOFs were applied as inhaled model particles for tracking. However, as *γ*‐CD‐MOFs are dissolvable in water,^[^ ^21^ ^]^ diphenyl carbonate (DPC) was used as a cross‐linkers to synthesize stable cross‐linked‐*γ*‐CD‐MOFs (CL‐MOFs).^[^ ^22^ ^]^ CL‐MOFs were then labelled with tracking molecules to harvest CL‐MOFs‐A488 and investigations were performed on these particles. The homogeneous cubic shape and limited geometric size of CL‐MOFs‐A488 contributed in the acquisition of reliable and repeatable results. The preferred aerodynamic size with high FPF value of 79.0 ± 2.8% and stable fluorescent intensity enabled the detection of deposited CL‐MOFs‐A488 in lungs. In this study, the purpose of the NGI experiment was to characterize the properties of powders rather than predicting the in vivo distribution results. As the NGI results (FPF) generally considered for humans may not be applicable to mice and the inhalability of aerosols above 2 micron is very low for a mouse.

Till now, in pre‐clinical researches, the in vitro deposition analyses of the aerosolized particles were performed in NGI and ACI, which were designed to simulate human physiological conditions. The in vivo distribution evaluation was normally done in mice or rats using the IVIS. This means that there is still a gap between in vitro and in vivo evaluations. The long‐term goal of this research involves establishing the correlation between particles properties in vitro and the deposition behavior in vivo and filling the gap between in vitro and in vivo analyzing results. In this study, we emphasize on coining a novel approach to reveal the inhaled particles distribution at 3D, in‐situ, and single‐particle level. Based on the established method, the accurate spatial distribution pattern in vivo can be generated. Then, on top of these results, the effects of aerodynamic size as well as the aerosolized ways on particles distribution can be investigated in the future. In addition, the in vitro simulation methods such as theoretical simulation, computational simulation, and even the NGI model can be optimized.

In addition, the aerosol size distribution of powder tested by the NGI using the gelatin capsule and dry powder inhaler may not be similar, when the particles are injected from the retention needle cap. Yet, the FPF is a choice for in vitro characterization of DPI. In this research, the in vivo experiments were performed in C57BL6 mice. This is not only for the suitable size of the animal, but also in light of that there are many mature transgenic disease models in C57BL6 mice for different pathological studies. However, there is not a standard method that is consistent with NGI for mice intrapulmonary administration yet. As aforementioned, the majority of preclinical researches utilized NGI to evaluate the aerodynamic size and deposition efficacy of powders in vitro and then applied retention needle caps to perform in vivo experiments in mice. However, the aerosol properties of particles generated by different devices varied. The potential future application of our method is to help establish the link between FPF from NGI and distributions in vivo and thus fill the gap between in vitro and in vivo results by investigating the distributional differences among particles with different FPFs.

Our approach has realized the displaying of inhaled particles spatial distribution at full volume scale with a resolution down to individual particles. And there are five notable features of inhaled particles’ distribution in the lung. 1) The majority of inhaled particles gathered on tracheal and bronchi surface. 2) The magnitude of particles deposited at upper part was markedly less than that at the middle and lower parts of the same lung lobe. 3) Particles tended to accumulate at the bifurcations of airways. 4) Particle clusters were also observed at the outlets of terminal bronchioles and the alveolar duct areas. 5) There were small aggregations or individual particles embedded in the interstitial space of humps on the surfaces of airways in all generations. Clearly, there were many single particles in air sacs, which were identified by the cubic shape. These various distribution patterns can be attributed to the particle properties and the unique anatomical structure of lungs. First, high superficial surface area of the micro‐size particles caused a tendency for some particle aggregation, and the size of such clusters, likely larger than 10 µm, would be deposited in/on the airways instead of alveolar areas.^[^ ^14^ ^]^ Second, the fluid dynamics determined by complex structural features of lungs plays an important role in the particles deposition and the airflow patterns in different areas of lungs may affect the region‐specific deposition of particles.^[^ ^23^ ^]^ Further, the direction of airflow changes at bifurcations, the inertia of the particles or aggregated particles would inhibit a change in direction of flow, resulting in a number of these particles depositing and adhering by impact and concentrating at forks in the airways. Meanwhile, the cilia structure on respiratory tracts could also obstruct the small aggregations or individual particles from entering alveoli. Finally yet importantly, the airflow pattern also had an impact on particles’ distribution. The sharp increase of cross‐sectional area in acini area caused a marked decreasing of airflow velocity,^[^ ^24^ ^]^ and the deposition of particles in the terminal bronchioles and alveoli may be caused of impaction and diffusion.^[^ ^25^ ^]^ Meanwhile, the aggregation of particles may have increased this trend.

The spatial distribution pattern provided an explanation why, in many cases the good fine particle deposition concluded from the in vitro NGI model do not correlate with in vivo IVIS data, and do not guarantee therapeutic efficacy. For aerosolized drug applications, a routine procedure in the exploration of inhaled particles’ engineering is utilizing NGI to evaluate the particles’ aerodynamic size in vitro followed by IVIS to assess the deposition of particles in vivo. In particular, it is generally regarded that intrapulmonary administration provides a targeted effect on pulmonary diseases. However, with the FPF value of 79.0±2.8% measured by NGI and fine distribution detected by IVIS, it has been found that the CL‐MOFs‐A488 particles were attached on the airways walls at all generations and the percentage of particles deposited in alveoli was 18.6%. This behavior is undesirable when considering the delivery of drug particles that are required to enter the alveoli for local or efficient cellular uptake for systemic treatment. For example, the pathogenic sites are mainly concentrated in the pulmonary acinar and alveolar duct areas for lung adenocarcinoma and pulmonary fibrosis. If the inhaled particles accumulate on lung airways surfaces, the therapeutic effects will be impacted negatively as only those particles deposited in the alveoli or lesion sites will deliver therapeutic benefits. However, the aggregation on airways walls would be beneficial to drugs exerting therapeutic actions in respiratory tracts such as bronchodilators. As the *β*2 receptors were found in higher expression throughout bronchi and bronchioles. Also, the deposited particles in each lung lobes were different, and almost half of particles deposited at the at the right caudal lobe. This means that the lung structure and airflow distribution play an important role in particles distribution. Additionally, this spatial distribution differentiation would be significant in human lungs since mouse lung is mostly monopodial in nature while humans have bipodial airway bifurcations.^[^ ^26^ ^]^ Overall, full understanding of particle distribution linked to lung architectures, together with detail of the target regions of the lungs required for specific therapeutic moieties is required to enable optimized drug efficacy when delivered by DPI. It is not sufficient to demonstrate the aerosolized particles’ distributions via NGI together with IVIS when designing DPI drug delivery systems.

As for the limitation of this method, on the one hand, it is not capable of achieving the dynamic observation of the particles deposition process in vivo as the imaging was performed after dissection and tissue process. On the other hand, the cross‐scale from micron to centimeter with high voxel resolution imaging generates huge database, which poses a challenge for storing, processing and analyzing. For each lung, more than 800 000 mosaics up to 20 terabytes were obtained. However, through this approach, the 3D lung architectures and the particles distribution patterns at single particle level were obtained with the highest resolution till now. This is also the first time to detect the intact distribution information of inhaled particles from trachea to alveoli. And owing greatly to the investigation of airway tree and deposited particles in a manner of large scale and high resolution, the region‐specific distribution patterns and structure‐associated deposition of the inhalable particles in lung were elucidated unprecedentedly.

In summary, this study has acquired the multiscale coreconstruction of lung architectures and the precise localizations of inhaled particles in lungs, which provides new detail and understanding of the in vivo spatial distribution of solid micron‐sized particles. The MOST system with Nissl‐staining was feasible for lung structure rendering, demonstrating potential application for other tissues such as heart, liver, spleen, kidney, tumor, and their pathological characterizations. Importantly, the data collecting and analyzing pipeline will make a great contribution on the particles distribution assessment and estimation of treatment effects for drug delivery systems.

## 4 Experimental Section

##### Materials and Cells

*γ*‐cyclodextrin (γ‐CD) was obtained from MaxDragon Biochem Ltd. (Guangzhou, Guangdong, China). DPC (99%) was purchased from Aladdin Reagent Co., Ltd. (Shanghai, China). Potassium hydroxide (KOH), polyethylene glycol 20000 (PEG 20000), triethylamine (TEA), *N*,*N*‐dimethylformamide (DMF), methanol, ethanol, acetonitrile, acetone, and other reagents of analytical grade were supplied by Sinopharm Chemical Reagent Co., Ltd. (Shanghai, China). Glycol methacrylate (GMA) series (Ted Pella Inc., Redding, CA, USA). Mouse IL‐6 Valukine ELISA Kit (VAL604), Mouse TNF‐*α* Valukine ELISA Kit (VAL609), and Mouse IL‐1*β* Valukine ELISA Kit (VAL601) were obtained from Bio‐Techne China Co.Ltd. Alexa fluor 488‐NHS was supplied by ThermoFisher Co., Ltd (Shanghai, China), CCK‐8 was provided by Dalian Meilun Biotechnology Co., Ltd. (Dalian, China). LDH Kit (C0017), Hoechst 33342 (C1022), and DiI (C1036) were obtained from Shanghai biyuntian biotechnology Co., Ltd. (Shanghai, China). Lipopolysaccharides (L2630‐10MG) were purchased from Sigma‐Aldrich (St. Louis, Missouri, USA). All reagents were commercially available and used as received. The cell lines of WI26‐VA4, MHS were purchased from ATCC (Manassas, VA 20108 USA). Cells were cultured in standard conditions by using EMEM (ATCC, Manassas, VA 20108 USA) and RPMI 1640 (Dalian Meilun Biotechnology Co., Ltd., Dalian, China), respectively. A549 and Calu‐3 were obtained from the Chinese Academy of Science Type Research Products (Shanghai, China) and cultured by RPMI 1640 and MEM (Dalian Meilun Biotechnology Co., Ltd., Dalian, China) medium, respectively.

##### Animals

Male C57BL6 mice (20 g) were provided by Shanghai Ling Chang Biotechnology Co., Ltd. All animal experiments were conducted according to the regulations of Shanghai institute of pharmacology, Chinese academy of sciences, and the IACUC number of the applied animal was No. 2018‐12‐zjw‐21.

##### Synthesis of Cuboidal Cross‐Linked γ‐Cyclodextrin Metal‐Organic Frameworks‐A488 as Inhalable Particles

Cuboidal CD‐MOF particles were synthesized following the method as reported.^[^ ^21^, ^27^ ^]^ Then, CL‐MOF particles were fabricated using CD‐MOF crystals as templates.^[^ ^22^ ^]^ Finally, Alexa fluor 488‐NHS was used to label the CL‐MOFs. Namely, Alexa fluor 488‐NHS (0.5 mg) and CL‐MOFs (500 mg) nanoparticles in DMF (8 mL) were stirred for 32 h at 37 °C in the dark. TEA (42 µL) was added to accelerate the reaction. The Alex fluorescence 488‐labelled cross‐linked γ‐cyclodextrin metal‐organic frameworks (CL‐MOFs‐A488) precipitates were washed with DMF and pure water and dried by freeze dryer.

##### Morphology and Size Characterization of Cross‐Linked *γ*‐Cyclodextrin Metal‐Organic Frameworks‐A488

SEM was utilized to characterize the morphology and particle size of CL‐MOFs‐A488. In brief, the CL‐MOFs‐A488 powder was dispersed on conducting adhesive and sputter coated with gold. Then, the samples were characterized by SEM (FlexSEM 1000, HITACHI). The distribution of particles size was measured and counted by Image pro plus. Hydrodynamic size and Zeta‐potential of CL‐MOFs‐A488 were confirmed by Zetasizer (Nano ZS; Malvern Instruments, Malvern, UK).

##### In vitro Deposition Analysis of Cross‐Linked *γ*‐Cyclodextrin Metal‐Organic Frameworks‐A488

In vitro deposition evaluation of the CL‐MOFs‐A488 was determined by NGI (NGI‐094, Copley Scientific Limited, Nottingham, UK). Simply, CL‐MOFs‐A488 (1 mg) particles were filled into hard gelatin capsules and aerosolized using the DPI device. Aerosols were produced at the air flow rate of 65 L min^−1^ with a run time of 3.7 s. The aerodynamic cutoff diameter of NGI stages 1–7 segments were 7.73, 4.20, 2.68, 1.63, 0.90, 0.52, and 0.32 µm, respectively, according to USP 601. Before testing, impactor stages were covered with 1% dimethicone *n*‐hexane solvent to trap the particles on the surface of stages. The particles deposited on the stages were collected using absolute ethyl alcohol followed by centrifugation. The sediments were suspended by PBS (200 µL), and the suspensions were transported to 96‐well plate. Then, the total fluorescent intensity at each stage was measured by IVIS. Finally, the FPF, MMAD, and GSD were calculated by Copley Inhaler Testing Data Analysis Software (Coply CITDAS, Version 3.10 Wibu). Three groups were tested and each group contained five capsules. To confirm the links between the mass and fluorescence intensity, the PBS solutions containing a series mass of CL‐MOFs‐A488 (10000, 5000, 2000, 1000, 500, 200, 100, 50, 20, 10, 0 µg mL^−1^) were prepared to evaluate the connection between the mass and fluorescence intensity. The mixtures (200 µL) were transferred to a 96‐well plate and the florescent intensities were detected by IVIS.

##### In vitro and In Vivo Stability of Cross‐Linked *γ*‐Cyclodextrin Metal‐Organic Frameworks‐A488

In vitro stability of CL‐MOFs‐A488 was investigated in the presence of PBS, plasma, artificial mucus, and absolute ethyl alcohol, respectively. In brief, CL‐MOFs‐A488 (50 µg mL^−1^) were incubated in a glass bottom dish with different medium with the stirring speed of 50 rpm at 37 °C for 7 days. At different time points, the morphological features were investigated by LSCM. To explore the fluorescent stability, CL‐MOFs‐A488 particles (500 µg mL^−1^, 200 µL per well) were incubated in a 96‐well plate at the same condition as mentioned above for 7 days. The changes of fluorescent intensity were detected by in vivo image system. For in vivo stability investigation, CL‐MOFs‐A488 (1 mg) was delivered into mouse lungs by endotracheal intubation. Then, the lung was excised at different time points and in vivo image system was applied to mirror the variation of fluorescent intensity.

##### Thermal Analysis

TGA with a thermal analysis system (NETZSCH 209 F3 240‐20‐382‐L, USA) was performed. Samples were weighed in an aluminum crucible and the percentage weight loss of the samples was monitored from 20 to 600 °C at a heating rate of 10°C min^−1^ under nitrogen.

##### Cytotoxicity Analysis of Cross‐Linked *γ*‐Cyclodextrin Metal‐Organic Frameworks‐A488

CCK‐8 was used to investigate the cytotoxicity of CL‐MOFs‐A488. Briefly, A549 Cells, WI26‐VA4 cells, MHS cells, and Calu‐3 cells were separately seeded into 96‐well plates at and maintained in a humidified incubator with 95% air and 5% CO_2_ at 37 °C. After being incubated overnight, a series of particle solutions (200 µL) were added to the medium and incubated for 24 h. Then, CCK‐8 solution (10 µL) was added to each well and incubated for 2–4 h, and the absorbance was measured at 450 nm using a microplate reader (Multiskan GO, Th dermo Fisher). Cell viability (%) = (A _sample_‐A _blank_)/(A _control_‐A _blank_) × 100

LDH release assay kit was applied to test the influence of CL‐MOFs‐A488 on cells’ membrane. Based on the manufacturers’ instructions, CL‐MOFs‐A488 with different concentrations was incubated with cells at 37 °C for 24 h. Then the 96‐well plate was centrifuged at 1000 rpm for 5 min and the supernatant was transferred to a new 96‐well plate. Then the supernatant were centrifuged at 3000 rpm for 10 min to remove the residual particles in medium. Supernatant (120 µL) was transferred to a new 96‐well plate and added with LDH detection solution (60 µL). After incubating at shaker at100 rpm for 30 min (25 °C), the absorbance of each well was detected by microplate reader (Multiskan GO, Th dermo Fisher) at 490 nm wavelength. Non‐treated cells were used as a control and LDH releases reagents‐treated cells were used as a positive control. Relative LDH release = (A _sample_‐A _blank_)/(A _control_‐A _blank_) × 100

##### Cellular Uptakes of Cross‐Linked *γ*‐Cyclodextrin Metal‐Organic Frameworks‐A488

Cells were cultured in a sterile glass bottom dish and incubated with CL‐MOF dispersions (50 µg mL^−1^) at 37 °C for 24 h. After incubations, cells were washed with PBS for three times to eliminate the adsorption of CL‐MOFs‐A488 on cell surface. Then, the CL‐MOFs‐A488‐incubated cells were stained with DiI and Hoechst 33342 for the labeling of lipid bilayers and nucleus respectively. After washing with PBS, the obtained samples were detected with CLSM technology (Leica, TCS, SP8, Germany). Each cell type has three dishes, and each dish was captured ten images at different regions. The cell numbers and the Florence area of particles were calculated by Image Pro software. The percentage of CL‐MOFs‐A488‐uptake cells (%) = (The number of cells with fluorescence‐labelled particles/The total number of cells) × 100; The green fluorescence area of single cell = The total area of green fluorescence/The number of cells with fluorescence‐labelled particles

##### Extracellular Pro‐Inflammatory Factors (IL‐6, IL‐1*β*, TNF‐*α*) Assay

Extracellular pro‐inflammatory factors (IL‐6, IL‐1*β*, TNF‐*α*) were determined via ELISA kits based on the manufacturers’ instruction. Briefly, MHS cells were incubated with CL‐MOFs‐A488 at different concentrations at 37 °C for 12 h. The culture mediums after incubations (100 µL) were obtained and transferred to ELISA plate. After incubations at 25 °C for 2 h, each well was washed with washing buffer. Then, the mouse conjugate (100 µL) was added to each well for 2 h at room temperature. After washing carefully, the plate was finally incubated with substrate solution (100 µL) for 30 min at room temperature and blocked by adding stop reagents. The absorbance of sample was detected by microplate reader (Multiskan GO, Th dermo Fisher) at 450 nm wavelength.

##### Histology and Immunofluorescence

Lung tissue sections stained with hematoxylin and eosin (H&E) and Nissl agents were utilized to observe the morphological features of lungs. Immunofluorescence was applied to investigate the CL‐MOFs‐A488 uptake by MHS. Briefly, Male C57BL6 mice were anesthetized followed by excising the lung. Then, the lung tissue was processed according to Histology and Immunofluorescence staining protocol, which was carried out by Wuhan Servicebio Technology Co., Ltd (Wuhan, Hubei, China). The data analysis was completed by Image Pro software.

##### Lung Tissue Preparation and Imaging by Micro Optical Sectioning Tomography System

The lung tissue for MOST system was prepared by a whole‐brain Nissl‐staining method.^[^ ^17^, ^28^ ^]^ The mouse lung was extracted after cardiac perfusion with phosphate‐buffered saline and 4% paraformaldehyde. Followed by Nissl staining and dehydration, the resin‐embedded mouse‐lung was fixed on a base for imaging by MOST system (Wuhan OE‐Bio Co., Ltd, Wuhan, China). Moreover, the raw dataset was acquired with the voxel size of 0.35 µm × 0.35 µm × 1 µm. The acquisition of entire lung sample generated about 1.5 terabytes raw image tiles.

##### Administration of Cross‐Linked *γ*‐Cyclodextrin Metal‐Organic Frameworks‐A488 Particles to Mice Lungs

The dry powder inhaling methods in mice were carried out as reported.^[^ ^29^ ^]^ Before performing animal experiments, the delivered dosage percentage of the aerosolized devices (injection syringe and retention needle cap) was investigated. Briefly, the weighted particles (M0) was added into the retention needle cap. The mass of retention needle caps before and after jetting were remarked as M1 and M2, respectively. The net delivered particles percentage = (M1−M2)/M0 × 100. Then, male C57BL6 mice (20 g) were anesthetized by intraperitoneal injection of 1% pentobarbital sodium solution. CL‐MOFs‐A488 (1 mg) was added into the retention needle cap. The otoscope was used to observe the airway of mice. Then the retention needle was inserted into the airway, and 1 mL of air was injected into the lungs of mice through a syringe.

##### Lung Tissue Preparation and Imaging by Fluorescence‐Micro‐Optical Sectioning Tomography System

All histological procedures referenced the preparation method of whole mouse brain samples.^[^ ^30^ ^]^ 5 min after lung administration, the anaesthetized mice were intracardially perfused with 0.01 m PBS and subsequent 4% paraformaldehyde. The lungs were carefully removed and post‐fixed in 4% paraformaldehyde at 4 °C. After post‐fixation for at least 24 h, each intact lung was rinsed for 24 h at 4 °C in 0.01 m PBS. Then, the lungs were dehydrated in a series of ethanol solutions (50%, 70%, and 95%) and then immersed in a series of GMA resin (Ted Pella Inc.,CA, USA) of increasing concentrations until 100% (70%, 85%, and 100% GMA containing 0.2% Sudan Black B). Subsequently, the tissues were immerged in adequate pre‐polymerized GMA solution for at least 3 days at 4 °C. Finally, the samples were embedded and polymerized in a vacuum oven at 50 °C for 36 h. The formula of GMA solution was in accordance with the previous described methods.^[^ ^30^, ^31^ ^]^

The prepared‐samples were glued to a metal base. Whole‐lung imaging was performed on f‐MOST system (Wuhan OE‐Bio Co., Ltd, Wuhan, China) with samples immersing in a water bath containing PI and Na_2_CO_3_ solution. In brief, the imaging parameters were set to obtain a high‐quality image. Then, the imaging and sectioning were performed automatically by f‐MOST system to complete the lung‐wide acquisition. The surface layer of sample was imaged at resolution 0.325 µm × 0.325 µm × 2 µm and subsequently the surface layer was cut away by a diamond knife to expose a new imaging surface. The imaging and sectioning process were repeated again and again until the sample was completely collected. During imaging process, the fluorescence signals of Alexa fluor 488 and PI molecules were simultaneously detected using two cameras. Continuous data acquisition lasted for over two weeks and obtained a dataset included more than 800 000 mosaic images in 8000 layers.

##### 3D Reconstruction of the Whole Lung Image

The original image tiles obtained by MOST system were preprocessed to obtain successive coronal images, with the aim of seamless image stitching and luminance non‐uniformity correction and image defect removal. Subsequently, image optimization was performed to improve image quality further by means of background correction, noise reduction and contrast enhancement.^[^ ^17^, ^32^ ^]^ The high‐quality processed coronal stacks were suitable to carry out 3D reconstruction of lung structure. The Amira software was used to visualize the whole lung surface and its inner tracheal tree. Image preprocessing for f‐MOST generating mosaic images was performed for the two channels (Alexa fluor 488 and PI) by steps of seamless stitching and illumination correction. The corresponding algorithm was implemented in MATLAB platform and optimized in parallel for‐loop.^[^ ^30^, ^31^ ^]^ High‐end graphic workstation with 1TB memory and 2 Nvidia Quadro P6000 graphics card (working in NVLink model) was equipped in this compute‐intensive research. Commercially available software Amira (Version 2019.1) was utilized for the image processing and visualization of the whole lung surface and its inner tracheal tree. Preprocessed entire sections were imported into Amira as voxel data and visualized with volume rendering module. Direct volume rendering implemented hardware‐accelerated computing of the light emission and absorption of each point in a data volume, which provided the capacity to display complex structure and texture of 3D objects. For the analysis of air way, the inverted volume data of whole lung was performed 3D bilateral filter (with a kernel size of 11 × 11 × 11) first. This edge‐preserving smoothing filter removed the noise and enhanced the contrast between bronchioles and surrounding tissues. Then the tracheal tree was segmented on filtered data by region growing algorithm (in a grey value range from 110 to 255) with manually marked seed point. Finally, the tracheal tree was segmented and the ISO‐Surface (a triangular approximation of the interfaces of segmented object) model was generated and displayed. Furthermore, five composed lobes of the lung identified based on the anatomy knowledge and labeled manually with the aid of volume edit module in Amira. After threshold value segmentation, the volume of tissue, and the inhaled MOF particles in each individual lobe were calculated correspondingly. Movies were created with the Animation Director tool in Amira by organizing the event list in storyboard and editing key frames in the time line. Figures were generated by taking snapshot of viewer panel using different display modules.

##### Statistical Analysis

Statistical analyses were carried out by One‐way analysis of variance with Tukey's post‐test, using GraphPad Prism software, from a minimum of three independent replicate values. The confidence level was set as significant when *p* < 0.05 (*) and highly significant when *p* < 0.01 (**) and *p* < 0.001 (***). All results were presented as the mean ± s.d.

## Conflict of Interest

The authors declare no conflict of interest.

## Author Contributions

X.S. and X.Z. contributed equally to this work. H.J., J.Z., and X.Y. designed the research. X.S. and X.Z performed the experiments. X.Y. performed the structure rendering. X.S. completed the synthesis and characterization of CL‐MOFs‐A488. X.S. assisted by X.R. and H.S. performed the cell and animal experiments. X.S. and X.Z. performed the tissue preparation and data collecting via MOST and f‐MOST system. P.Y., Z.G., L.W., C.W., and X.Y. substantively revised the manuscript. All the authors discussed and prepared the manuscript.

## Supporting information

Supporting Information

Supplemental Movie 1

Supplemental Movie 2

Supplemental Movie 3
