# Supplementary material for: Multiscale Co‐reconstruction of Lung Architectures and Inhalable Materials Spatial Distribution
Source: Adv Sci (Weinh). 2021 Feb 8;8(8):2003941. doi: 10.1002/advs.202003941 (PMC8061354; doi:10.1002/advs.202003941)
Supplement: Supplementary file 1 — Supporting Information [file ADVS-8-2003941-s001.pdf]

© 2021 Wiley-VCH GmbH

ADVANCED SCIENCE
Open Access

Supporting Information

for *Adv. Sci.,* DOI: 10.1002/advs.202003941

Multiscale co-reconstruction of lung architectures and inhalable materials spatial distribution

Xian Sun, Xiaochuan Zhang, Xiaohong Ren, Hongyu Sun, Li Wu, Caifen Wang, Xiaohui Ye, Peter York, Zhaobing Gao, Hualiang Jiang, Jiwen Zhang*, Xianzhen Yin**

Supporting Information

Multiscale co-reconstruction of lung architectures and inhalable materials spatial distribution

Xian Sun, Xiaochuan Zhang, Xiaohong Ren, Hongyu Sun, Li Wu, Caifen Wang, Xiaohui Ye, Peter York, Zhaobing Gao, Hualiang Jiang, Jiwen Zhang*, Xianzhen Yin**

Corresponding Author:

Xianzhen Yin, Email: xzyin@simm.ac.cn;

Jiwen Zhang, Email: jwzhang@simm.ac.cn;

Hualiang Jiang, Email: hljiang@simm.ac.cn

This file includes:

Figure S1-S10

Captions for Movie S1-S3

Other Supplementary Material for this manuscript includes the following:

Movie S1-S3

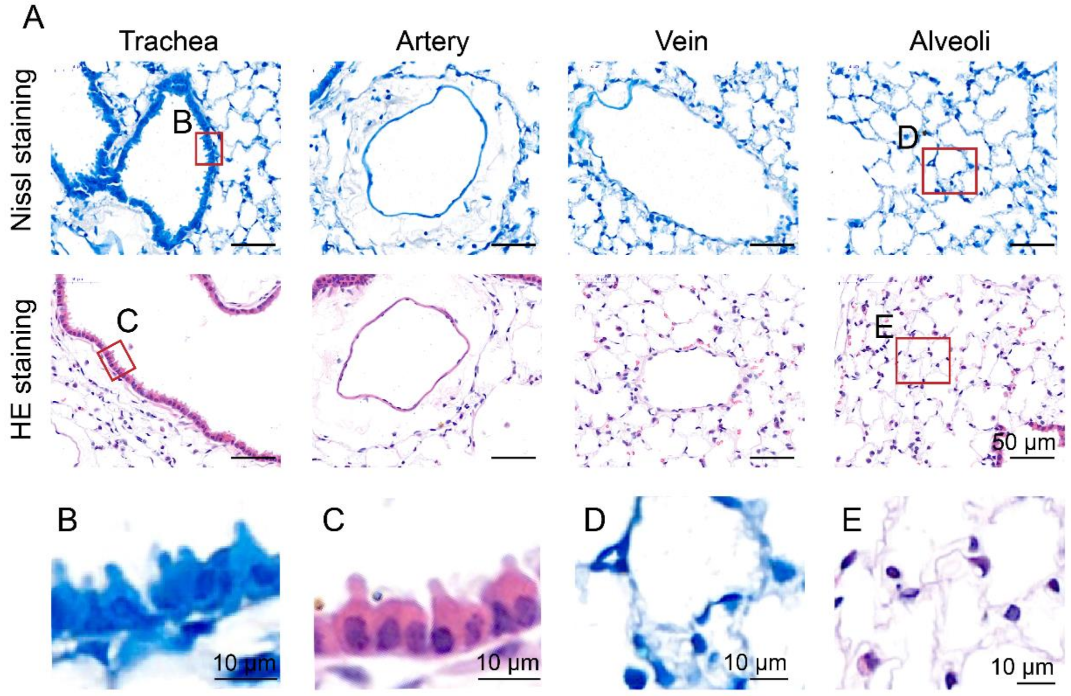

Figure S1. The lungs' slices from HE staining and Nissl staining. (A) The slice of four specific structures (trachea, arteries, veins and alveoli) stained by HE and Nissl, respectively. The bar was 500 μm. (B-E) The enlarged views corresponding to red boxes in (A).

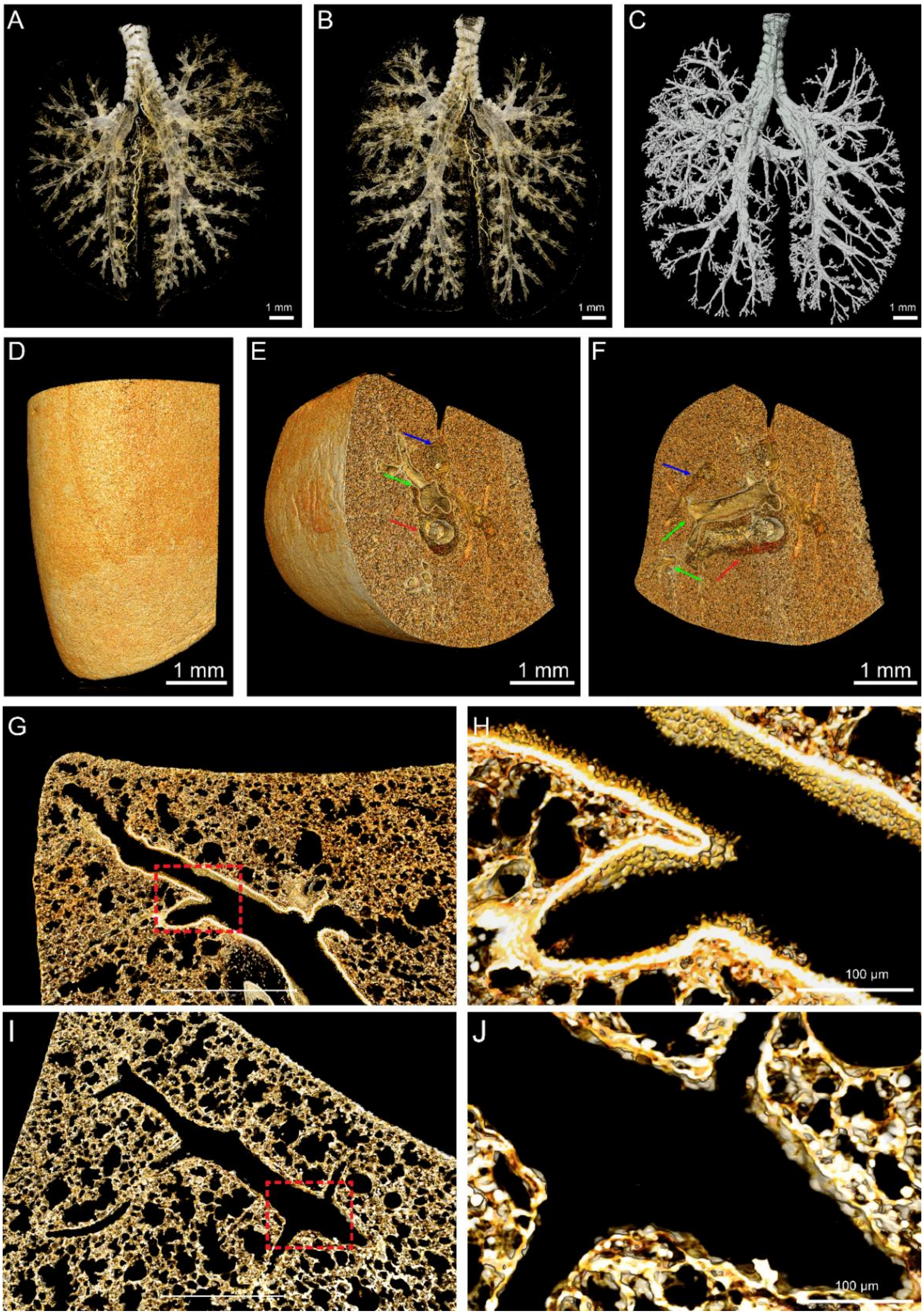

Figure S2. The rendering of lung based on Nissl staining. (A, B) The tracheal trees segmented by grey threshold. (C) The rendered airway tree terminated in alveolar duct according to airway tracking. (D-F) A portion of lung architectures including airways and vessels. The inner respiratory tracts (green arrows), veins (blue arrows) and arteries (red arrows) in a local region of lung. The surface of airways (G, H) and veins (I, J).

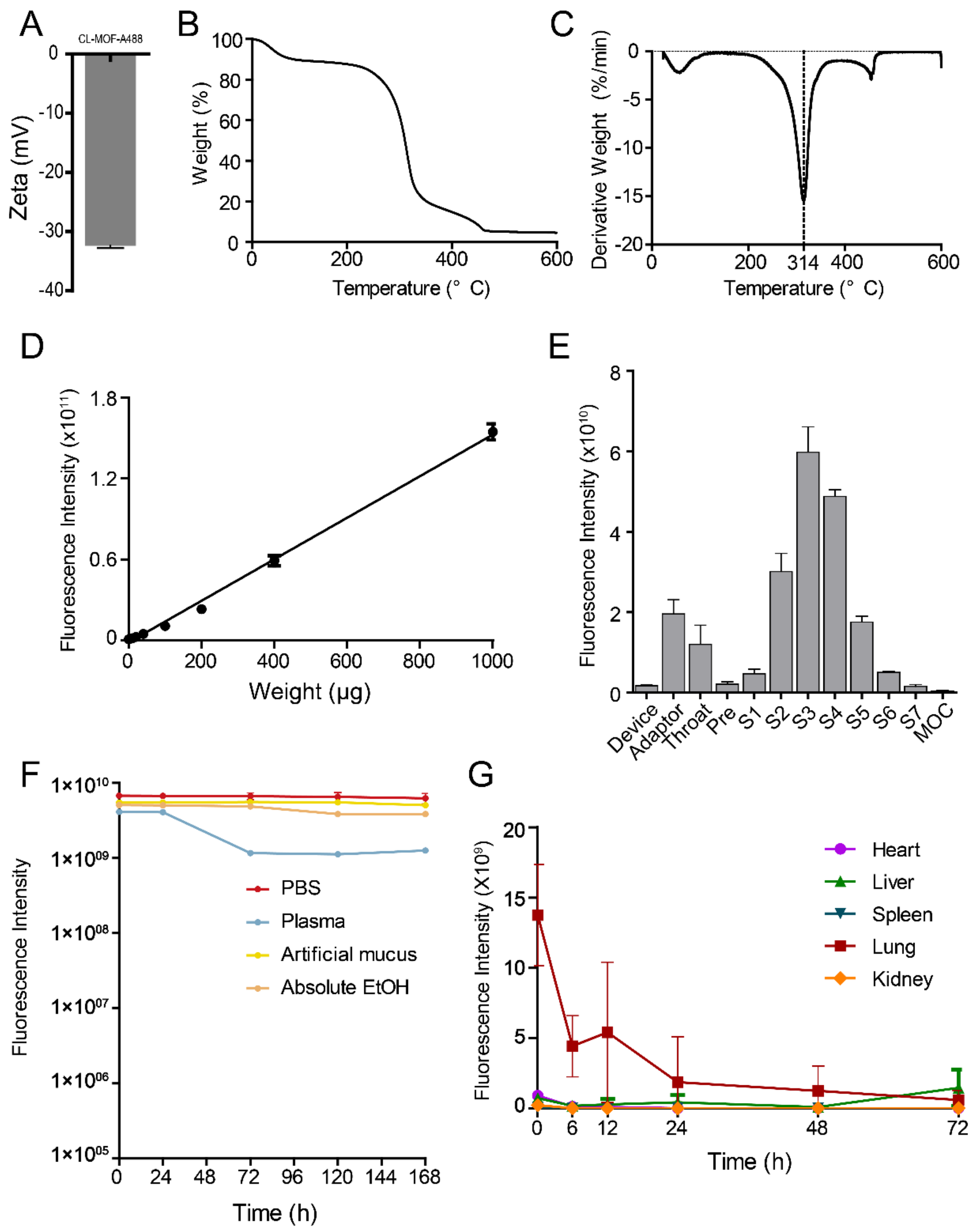

Figure S3. Characterizations of CL-MOFs-A488 for inhalation. (A) Zeta potential of CD-MOFs-A488 (n=3). (B) TGA profile of CL-MOFs-A488. (C) The derivative of TGA profile. (D) The linear regression between the mass and fluorescence intensity of CL-MOFs-A488. (r^2=0.9966, n=6). (E) The fluorescence intensity of CL-MOFs-A488 at different stages of NGI (n=3). (F) The fluorescent intensity of CL-MOFs-A488 in four mediums over 7 days (n=5). (G) CL-MOFs-A488 particles were delivered into mouse lungs by passive inhalation. The intensity of fluorescence signals collected by the IVIS (n=3). The data were processed by GraphPad Prism 5 and presented as means ± s.d.

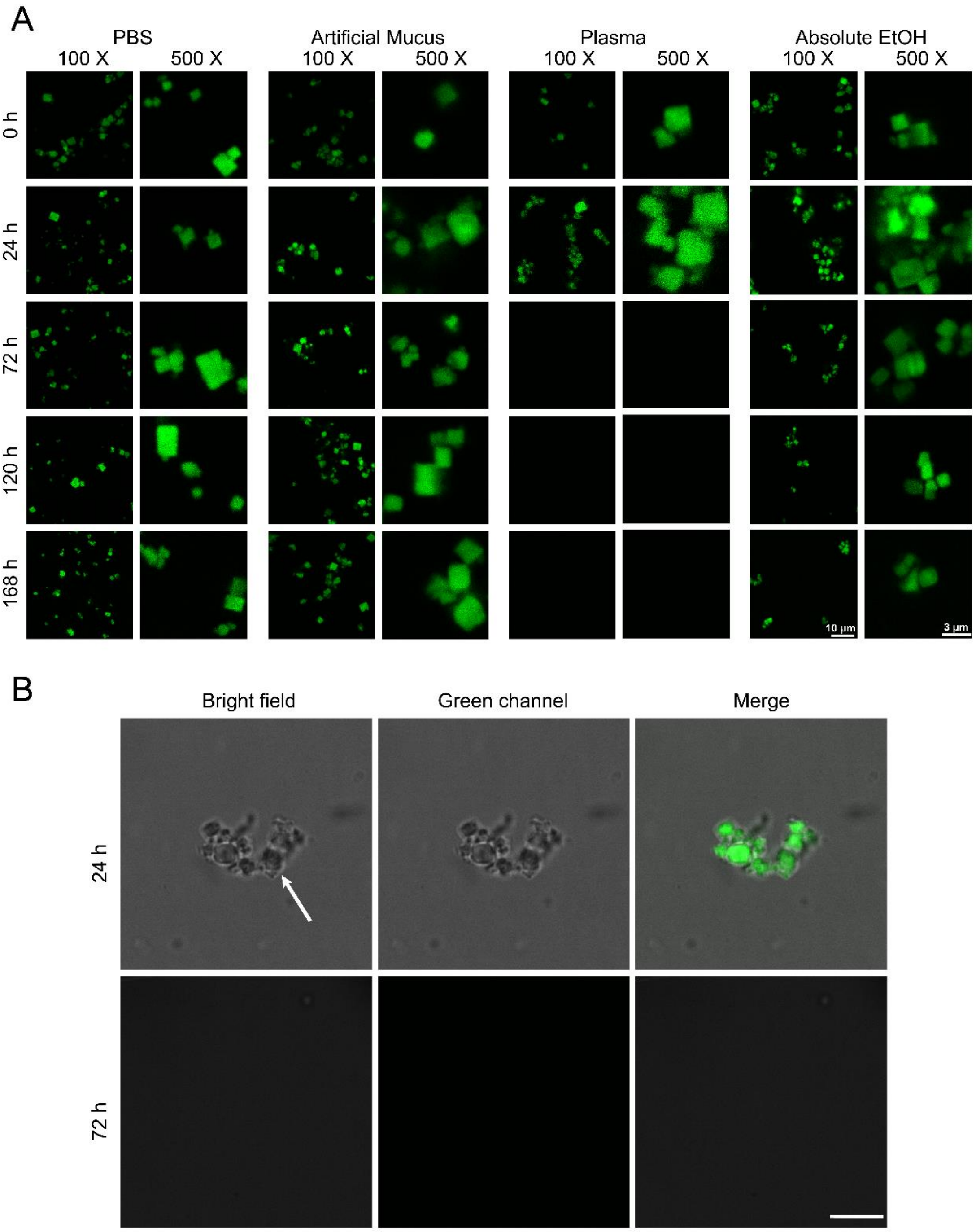

Figure S4. The morphological stability of CL-MOFs-A488 captured by LSCM. (A) In PBS, Artificial mucus and Absolut Ethyl alcohol medium, the fluorescent signals kept square shape over 7 days. The fluorescence along with cubic shape vanished at 72 h in Plasma. Scale bar for 100X: 10 µm, Scale bar for 500X: 3 µm. (B) CL-MOFs-A488 in Plasm for 24 h and 72 h. The bright fields showed the CL-MOFs-A488 particles (arrow). Green channels represented the florescent signals of CL-MOFs-A488 particles. Merged images indicated that the co-

localization of CL-MOFs-A488 particles and florescent signals. At 24 h, the CL-MOFs-A488 particles still kept cubic morphology with detectable florescent signals. At 72 h, the CL-MOFs-A488 particles disappeared together with florescent signals. The bar was 10 μm.

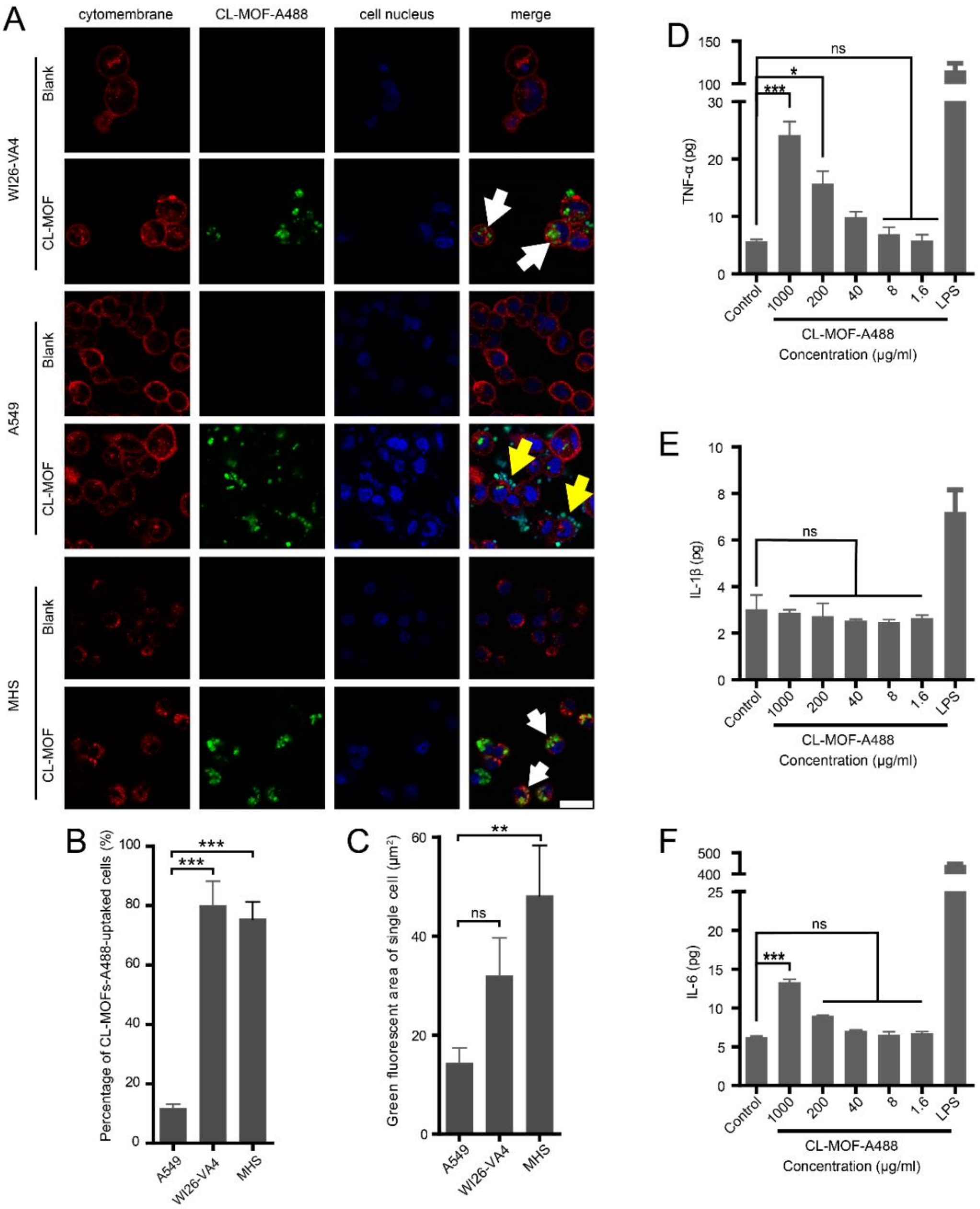

Figure S5. The internalization and Enzyme-linked immunosorbent assay (ELISA) of CL-MOFs-A488 in different cell lines. (A) Confocal images of cells after CL-MOFs-A488 incubations. The bar was 25 μm. (B) The percentage of CL-MOFs-A488-uptaked cells. (C) Green fluorescence area of single cell. (D-F) Extracellular primary pro-inflammatory factors (IL-6, IL-1β, TNF-α) induced by CL-MOFs-A488 were measured by ELISA kits (n=3). The data were expressed as means ± s.d. Statistical significances were calculated by one-way ANOVA test with Tukey's post-test: *p<0.05, **p<0.01, ***p<0.001, ns: no significant difference.

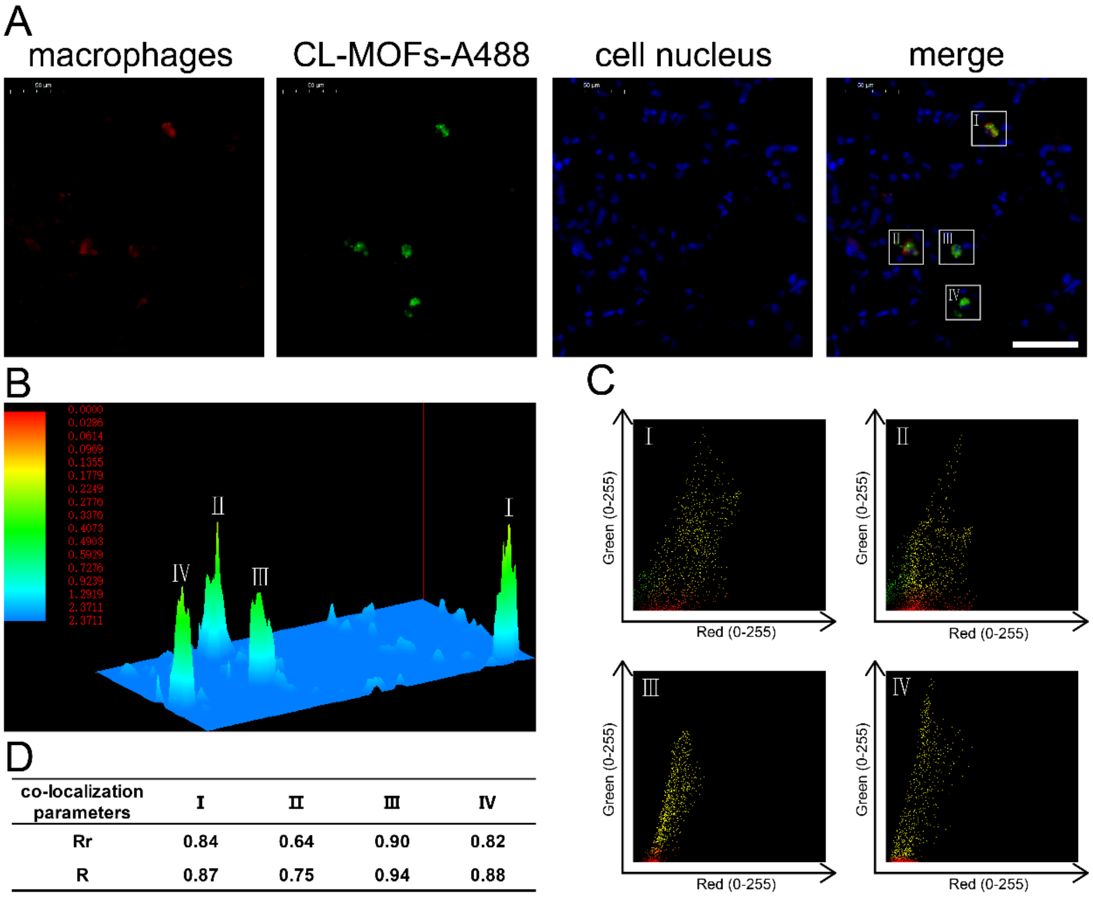

co-localization parameters	I	II	III	IV
Rr	0.84	0.64	0.90	0.82
R	0.87	0.75	0.94	0.88

Figure S6. The immunofluorescence analysis of CL-MOFs-A488 in alveolar macrophages. (A) F4/80 (red) was used to labeled alveolar macrophages specifically in mouse lungs. Blue channels: nucleus; red channels: alveolar macrophages; green channels: CL-MOFs-A488 particles. Four cells were circled separately for analysis. The bar was 50 μm. (B) The 3D view of color co-localization. The peaks represented the location that red color and green color existed simultaneously. (C) Color co-localization of four cells. X-axis represented the component from 0 to 255 of red, Y-axis represented the component from 0 to 255 of green. (D) Red-Green co-localization parameters of four cells. Rr: Pearson's correlation, R: Overlap coefficient. The data were calculated by Image Pro.

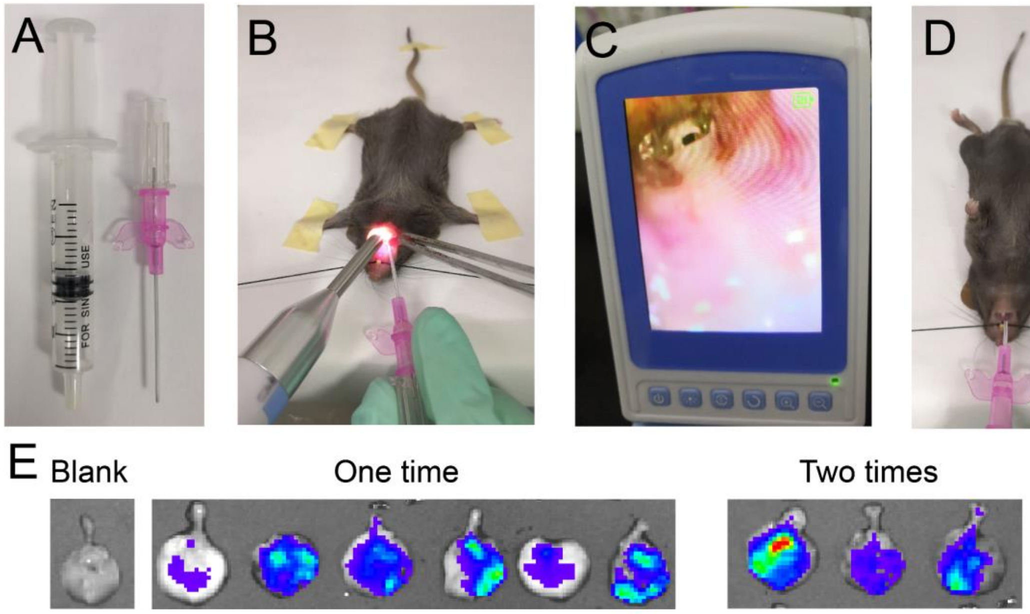

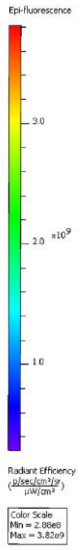

F

The times of spraying air	1mL Air each time	
	One time	Two times
Total	6	6
Dead	0	3
Alive but not sprayed	0	0

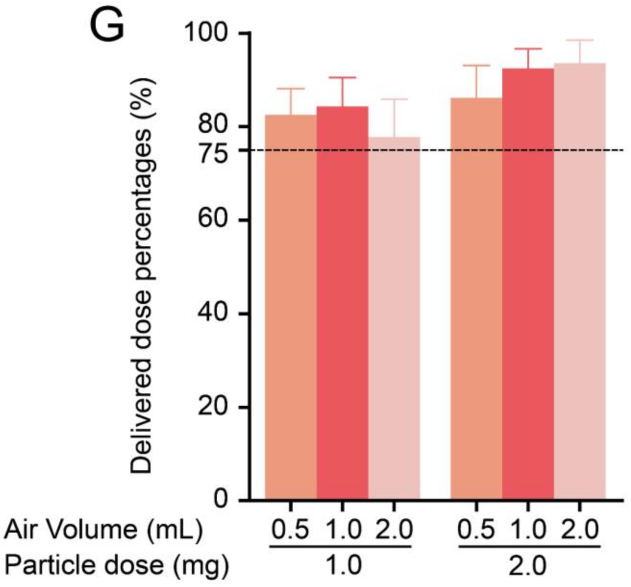

Figure S7. Investigations on the administration method of endotracheal intubation in mice

lungs. (A-D) Endotracheal intubation administration device for mouse lungs. To improve the success rate of endotracheal intubation in mice, the internal steel needle was retained when the powders was administered with the retention needle (A, B). An otoscope was used to visualize the location of the trachea (C). (E) The particles' distributions in mouse lungs detected by IVIS. (F) The success rate of administration and the survival status of mice with different air injection times (1 mL at a time, n = 6). The success rate of powder administration in mice using this method can reach 100%. One injection can ensure the survival state of mice. (G) The percentages of delivered dosage with different dosages (1 mg and 2 mg) and air volumes (0.5mL, 1 mL and 2 mL) (n=3). The data were expressed as means ± s.d. calculated by GraphPad Prism 5.

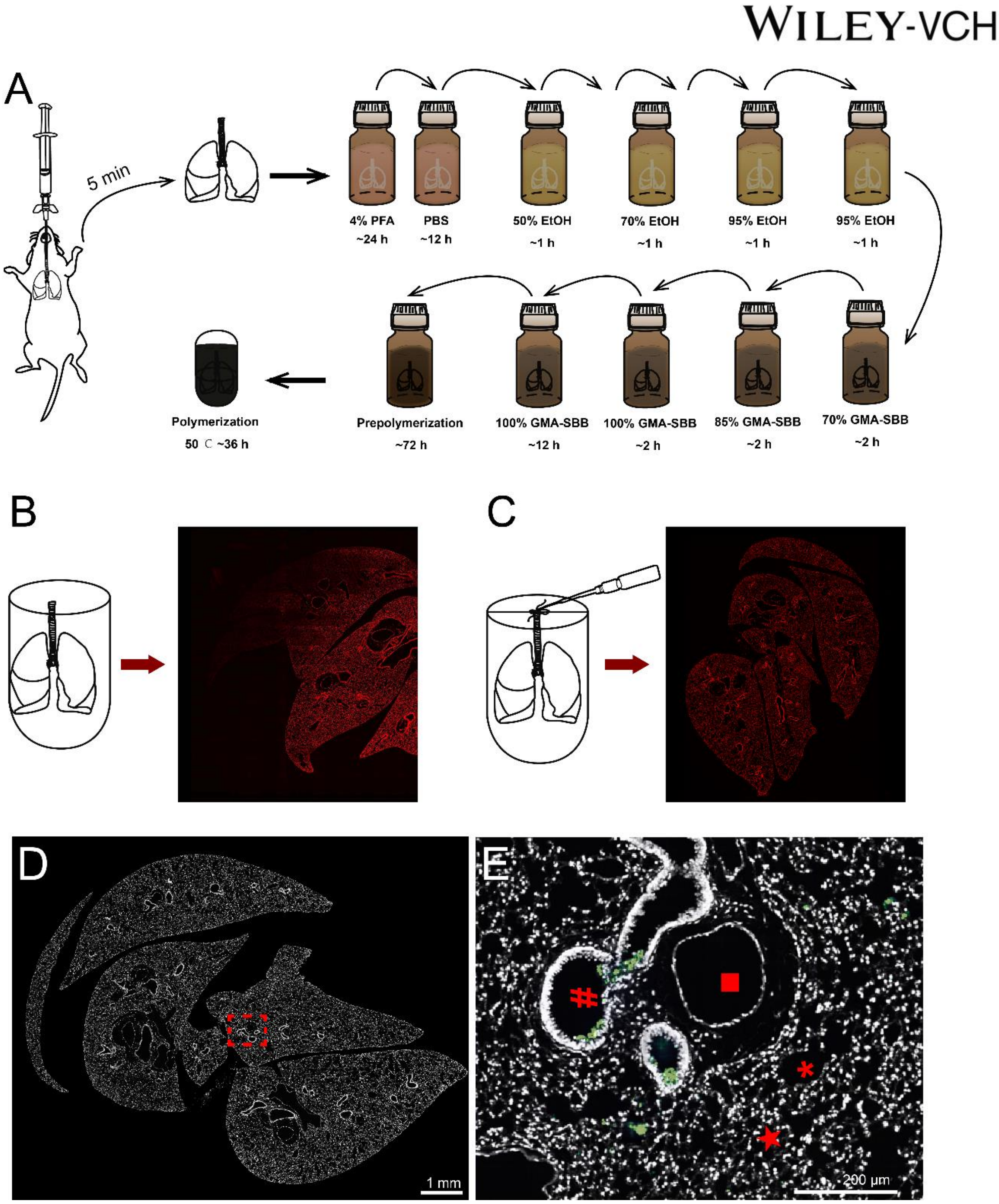

Figure S8. Lung tissue preparation and f-MOST imaging. (A) Schedule of lung tissue preparation (B) The image became fuzzy at the area of the lung attached to the edge of the capsule. (C) A short stick was used to keep the lung at the center of the capsule to keep images integrated. (D) The section obtained by HE staining. The bar was 100 μm. (E) The coronal image of lung. (F) The detail information of the red box in (E). Alveolar duct (*), Alveoli (★), Vessel (■), Bronchiole (#).

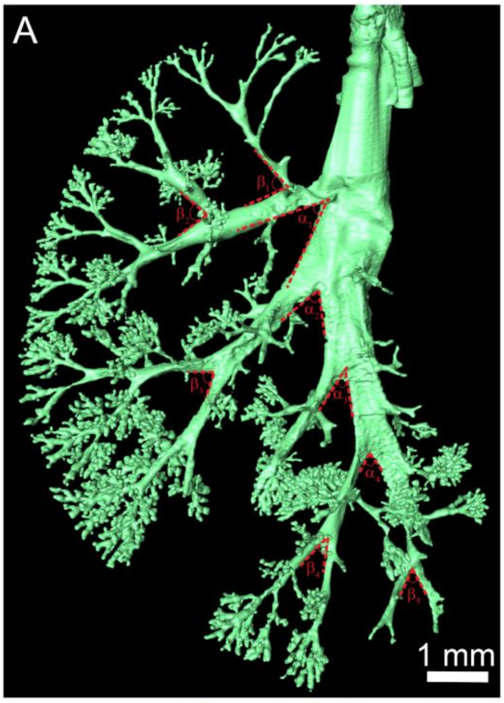

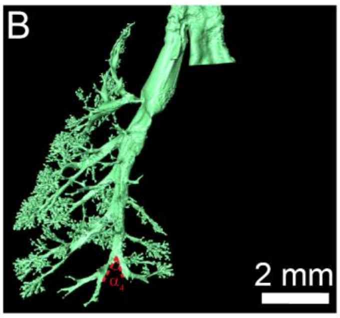

C

Angle	Value
α_1	47.7 °
α_2	58.2 °
α_3	41.3 °
α_4	64.1 °
α_5	46.1 °
β_1	70.9 °
β_2	76.4 °
β_3	72.6 °
β_4	34.8 °
β_5	53.0 °

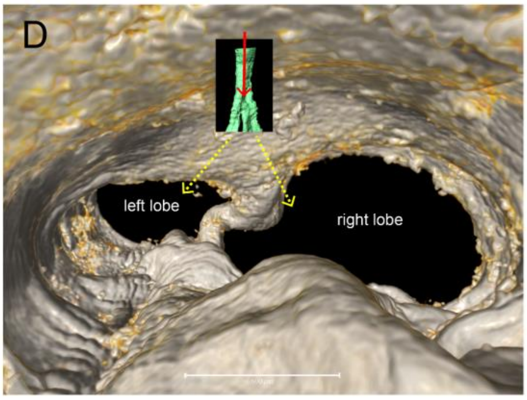

Figure S9. The angles among bronchi in left lobe. (A-B) The tracheal trees with different views. (C) The values of angles. (D) The endoscopic view of the extrapulmonary tracheas. The diameter of right bronchi was larger than that of left bronchi.

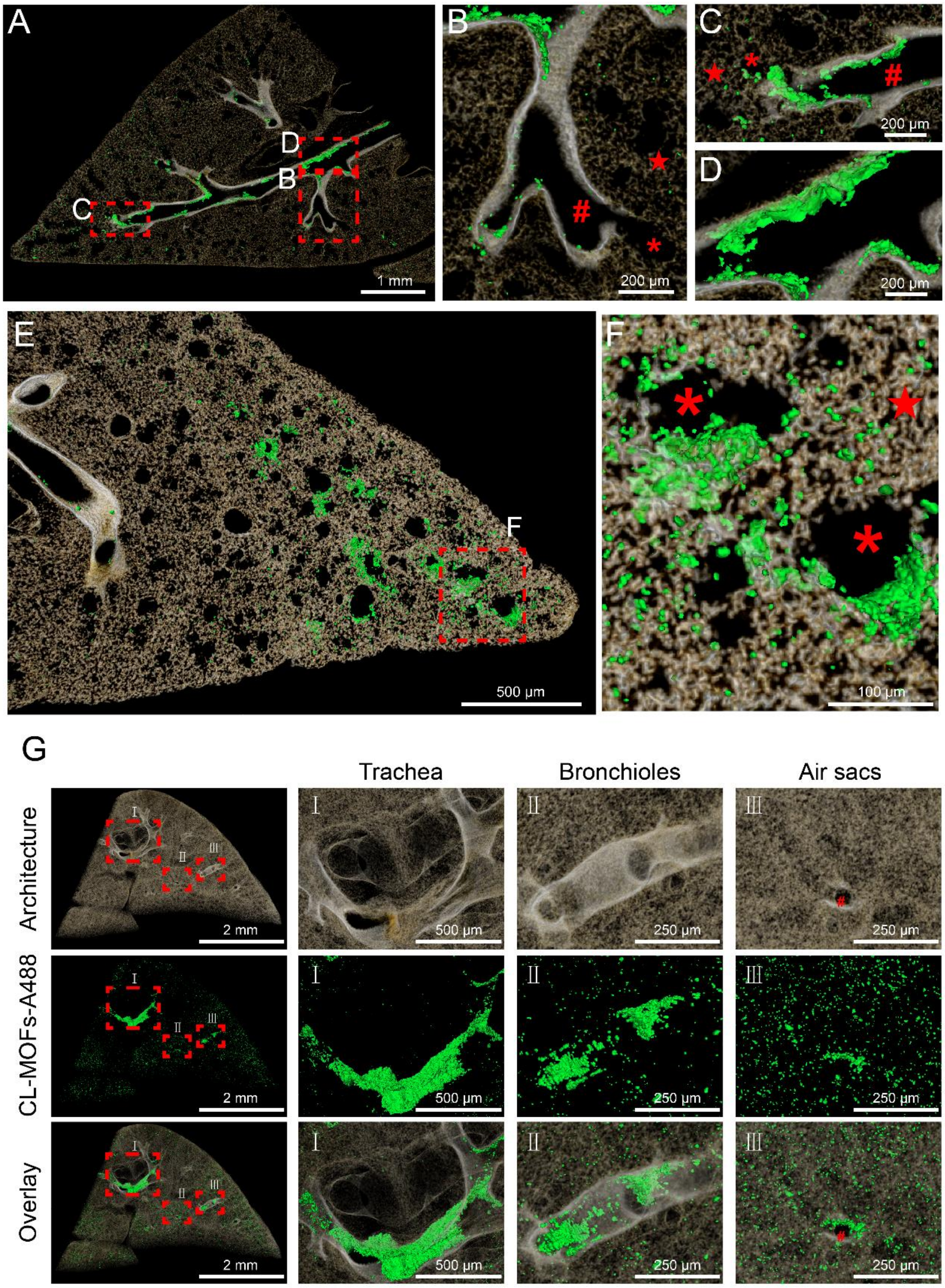

Figure S10. The existential state of CL-MOFs-A488 particles in mouse lung. (A) The projection of lung along the bronchiole. (B-D) The magnification of the areas marked with red dashed line in (A). (E) The projections of lung transecting the bronchus. (F) The detail information of inhaled particles in alveolar duct and alveoli enlarged from the red box in (E). (G) The distributional differences of CL-MOFs-A488 in trachea, bronchiole and air sacs. I:

trachea; Ⅱ: bronchiole; Ⅲ: air sacs. Boxed areas were shown at a higher magnification. The lung tissue was showed in transparent grey and the CL-MOFs-A488 particles were in green. Alveolar duct (*), Alveoli (★), Bronchiole (#).

Captions for Movie S1-S3

Movie S1. The whole lung structure

The coronal images created by the MOST system were overlaid to reconstruct the lung architectures. The trachea tree was extracted based on the diversity of grey levels and morphological features. The segmented airways were colored by different lung lobes and inner diameter, respectively.

Movie S2. The fine structure of mouse lungs

A portion of the lung containing bronchioles (green), arteries (red), veins (blue) and sponge-like acini area (yellow) was segmented. Endoscopic views manifested the surface differences between bronchioles and alveoli.

Movie S3. The spatial distribution of inhaled particles in whole-lung scale

The majority of particles were adhered on the surface of airway. The particles in bronchiole and alveoli were demonstrated by virtual endoscopy.
